# Daily full spectrum light exposure prevents food allergy-like allergic diarrhea by modulating vitamin D_3_ and microbiota composition

**DOI:** 10.1038/s41522-021-00213-8

**Published:** 2021-05-06

**Authors:** Po-Jung Chen, Toshiaki Nakano, Chia-Yun Lai, Kuei-Chen Chang, Chao-Long Chen, Shigeru Goto

**Affiliations:** 1 Graduate Institute of Clinical Medical Sciences, Chang Gung University College of Medicine, Kaohsiung, Taiwan; 2 Liver Transplantation Center, Kaohsiung Chang Gung Memorial Hospital, Kaohsiung, Taiwan; 3 Nobeoka Medical Check Center, Fukuoka Institution of Occupational Health, Nobeoka, Japan; 4 Faculty of Nursing, Josai International University, Togane, Japan

**Keywords:** Policy and public health in microbiology, Microbiota, Applied microbiology

## Abstract

The importance of sun exposure on human health is well recognized, and a recent trend in the avoidance of sun exposure has led to the risk of missing the beneficial effects such as vitamin D_3_ biogenesis. Vitamin D_3_ insufficiency is one of the risk factors for the development of food allergies (FAs), and vitamin D_3_ status controls gut homeostasis by modulating the microbiota. This study aimed to explore the impact of daily full spectrum light exposure (phototherapy) on the pathogenesis of FAs. Phototherapy ameliorated allergic diarrhea and improved FA-associated vitamin D_3_ insufficiency and dysbiosis. Fecal microbiota transplantation (FMT) of FA donor feces induced allergic diarrhea with OVA-specific IgE elevation in naïve mice. In contrast, FMT of naïve donor feces ameliorated allergic diarrhea in established FA mice, suggesting the involvement of the microbiota composition in FA. Phototherapy is an alternative approach for the prevention of FA-like allergic diarrhea through the modulation of vitamin D_3_ status and microbiota composition.

## Introduction

Food allergies (FAs) are common in children and adults, and the prevalence of FAs has increased dramatically in the past few decades in westernized countries^1^. FA is considered mainly an IgE-mediated type I (immediate) hypersensitivity, and the continuous sensitization of food allergens may cause mild-to-severe allergic reactions such as dermatitis, gastrointestinal and respiratory distress, and life-threatening anaphylaxis. The traditional treatment approach to FA is the avoidance of allergenic foods, and patients must carry a self-injectable form of epinephrine for emergency treatment of anaphylaxis^2^. In addition to the lack of a complete cure for FAs, the mechanisms of FAs are still uncertain.

There are several theories explaining the increase in FAs, including (1) changes in our food system (e.g., the introduction of genetically modified organisms, the addition of numerous chemicals to our food), (2) the hygiene hypothesis, (3) epigenetics, and (4) delayed allergen exposure^3–7^. In addition, epidemiological evidence has shown that low vitamin D_3_ levels in infancy appear to be a risk factor for the development of FAs^8^. Interestingly, pediatric FAs are more prevalent in regions farther away from the equator^9,10^. The negative correlation between the amount of sun exposure immediately after birth and food sensitization^11^ as well as the higher frequency of autumn–winter births in FA patients^12^ strongly suggest the significance of sun exposure and vitamin D_3_ generation in the prevention of FA. However, due to the destruction of the ozone layer in the past few decades^13^, we are exposed to large amounts of UVA and UVB, which cause skin aging, damage, and cancer progression^14^. Therefore, there have been many public health recommendations to avoid UV radiation exposure, which may cause significant harm to public health due to vitamin D_3_ insufficiency^15^.

Since Niels Ryberg Finsen (1860–1904) introduced full spectrum light therapy (short phototherapy) for the treatment of skin disease^16^, phototherapy has been applied for many diseases, such as mental disorders^17,18^, sleep disorders^19,20^, neurological disorders^21,22^, and neonatal jaundice^23^. In our previous study, we demonstrated reduced expression of vitamin D_3_ in nonalcoholic steatohepatitis (NASH), and daily exposure to full spectrum light and vitamin D_3_ supplementation ameliorated NASH progression in rats^24^. This study is the first confirmation of the therapeutic potency of phototherapy and vitamin D_3_ supplementation in an animal model of NASH, which clearly builds the basis for subsequent human therapeutic trials for NASH (ClinicalTrials.gov identifier: NCT01571063)^25^. In addition to the clinical impact of phototherapy in terms of the elevation of vitamin D_3_ in NASH patients^26^, we have demonstrated the therapeutic potential of phototherapy in experimental models of colitis^27^ and peritonitis^28^. However, there are no studies applying phototherapy for the prevention of FA.

In addition to the association between vitamin D_3_ status and FAs, accumulating evidence suggests the impact of compositional and functional changes in gut microbiota on the dramatic increase in FA prevalence^29–31^. The gut microbiota is recognized as a target for preventive and therapeutic intervention against FAs^32^, while limited information is available addressing the therapeutic potential of fecal microbiota transplantation (FMT) for allergic disorders^33^. Vitamin D_3_ status controls gut homeostasis by modulating the gut microbiota^34^, and a recent study pointed to the modulation of gut microbiota by narrow band UVB light exposure^35^. However, there are no studies exploring the synergistic impact of full spectrum light on vitamin D_3_ status and microbiota composition. In this study, we explored the impact of phototherapy on the pathogenesis of OVA-induced FA in mice and discussed the significance of vitamin D_3_ status and the fecal microbiota composition in FA.

## Results

### Impact of phototherapy on the pathogenesis of FA

**Fig. 1:**
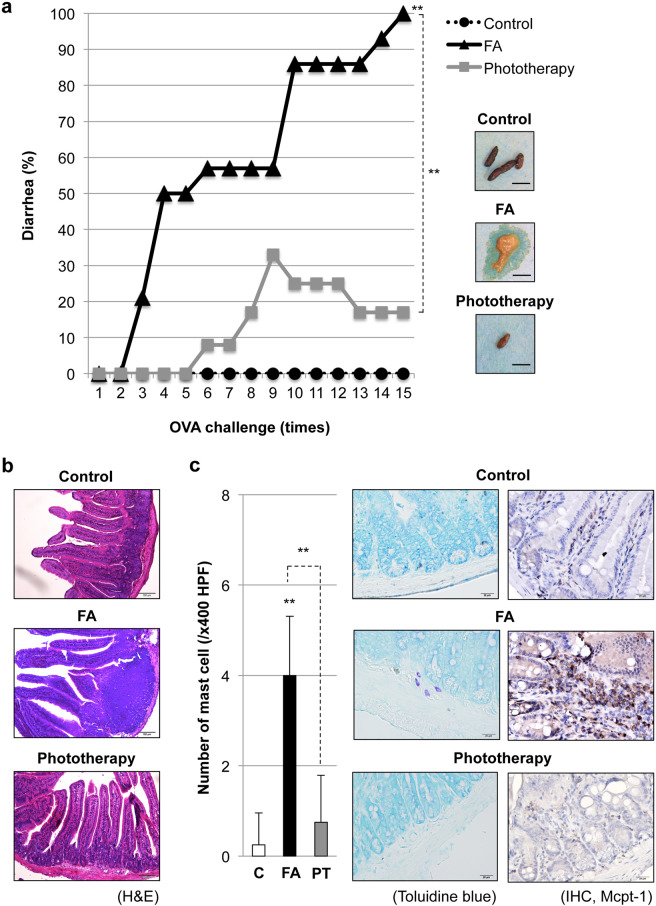
Impact of phototherapy on the pathogenesis of food allergy (FA). **a** Establishment of FA mice. Repeated intragastric (i.g.) OVA challenge was performed 15 times in OVA-sensitized BALB/c mice with (*n* = 12) or without phototherapy (*n* = 14). Naïve mice (*n* = 8) without OVA immunization were treated with PBS as a control. Allergic diarrhea was assessed visually by monitoring mice for 30 min after each i.g. exposure. **P < 0.01 vs. the control group or the indicated pair (Tukey’s multiple comparisons test). Enclosed pictures are representative feces after i.g. OVA challenge in the control, FA, and phototherapy groups. Scale bar: 5 mm. **b** Histological evaluation of the intestines in FA mice. The pictures at ×100 magnification are representative of at least six individuals in each group. H&E hematoxylin and eosin staining. Scale bar: 100 μm. **c** Mucosal mast cell activation in FA mice. Left: mast cells were visualized by toluidine blue staining and counted under a microscope at ×400 magnification. Values are presented as the mean ± SD of at least six individuals in each group. C control, FA food allergy, PT phototherapy. **P < 0.01 vs. the control or phototherapy group (Student’s *t* test). Right: immunohistochemistry (IHC) for mast cell protease 1 (Mcpt-1). The pictures at ×400 magnification are representative of at least six individuals in each group. Scale bar: 20 μm.

An OVA-induced FA model was established to explore whether phototherapy could prevent FA symptoms. As shown in Fig. 1a, allergic diarrhea was confirmed at the 3rd OVA challenge in OVA-sensitized FA mice. The incidence of allergic diarrhea gradually increased after the 3rd OVA challenge and all mice (14/14) showed allergic diarrhea at the 15th OVA challenge in the FA group. Histological data revealed mucosal and submucosal inflammation and shortening of intestinal villi in FA (Fig. 1b). On the other hand, phototherapy reduced the incidence of allergic diarrhea, and the maximum incidence of allergic diarrhea was 33.3% (4/12) at the 9th OVA challenge. The reduced incidence of allergic diarrhea at the 15th OVA challenge (2/12, 16.6%) with phototherapy suggested the potential effect of phototherapy on both the prevention and therapeutics of FA. Histological data clearly revealed the amelioration of allergic inflammation by phototherapy (Fig. 1b).

To further explore the impact of phototherapy on the pathogenesis of FA, we next evaluated FA-associated mucosal mast cell activation in the intestine after the 15th OVA challenge. As shown in Fig. 1c (left), we confirmed the FA-induced accumulation of mucosal mast cells in the intestinal submucosa and lamina propria, while phototherapy suppressed this accumulation. The elevation of mucosal mast cell protease 1 (Mcpt-1) at the mRNA (Supplementary Fig. 1a) and protein (Fig. 1c, right) levels was also observed, thus suggesting the activation and degranulation of intestinal mucosal mast cells during the development of FA. Furthermore, we confirmed systemic elevation of Mcpt-1 in FA, while phototherapy suppressed it (Supplementary Fig. 1b).

### Impact of phototherapy on immunoglobulin production in FA

**Fig. 2:**
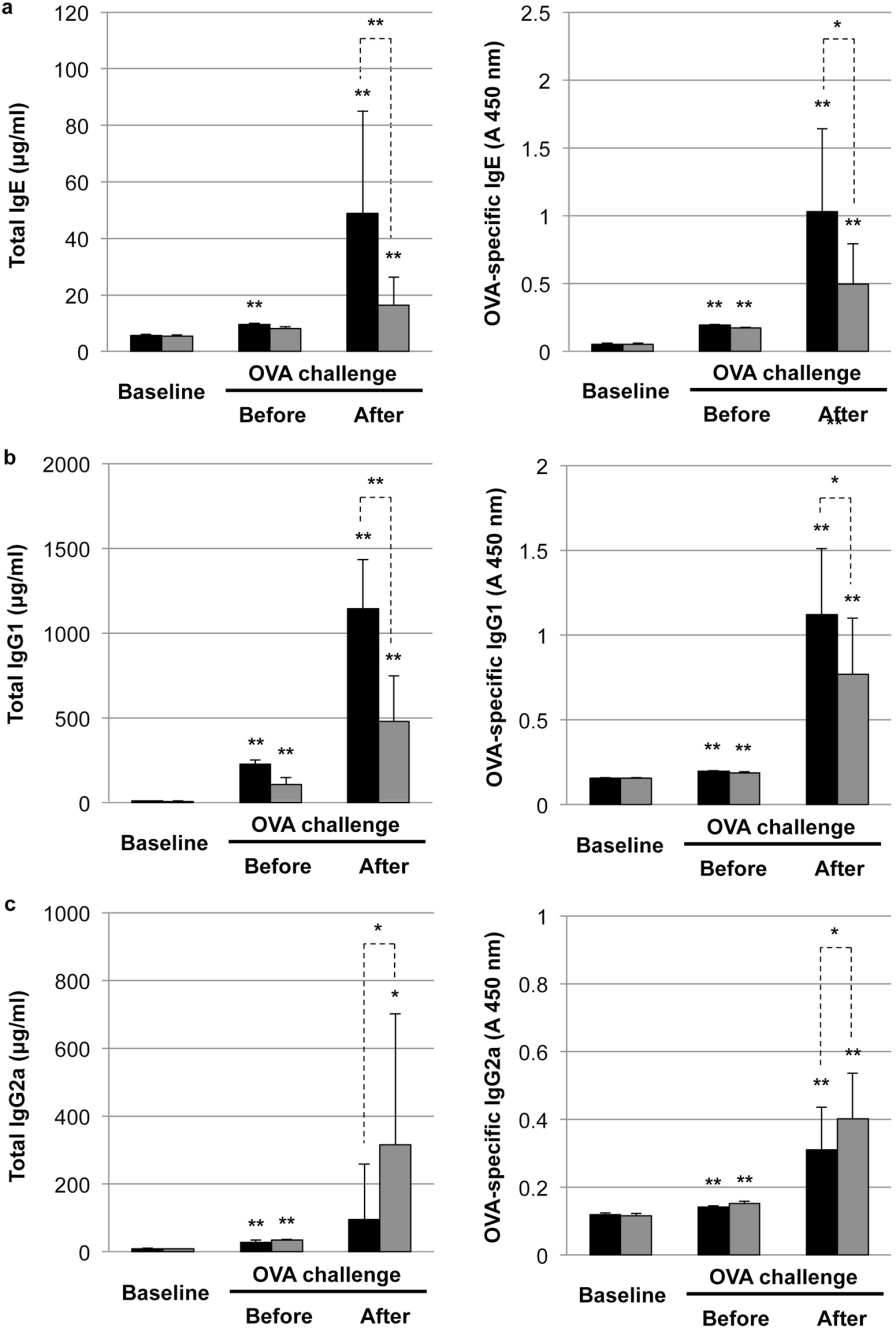
Impact of phototherapy on immunoglobulin production in food allergy (FA). Serum levels of total or OVA-specific IgE (**a**), IgG1 (**b**), and IgG2a (**c**) in the FA (black bar) and phototherapy (gray bar) group were measured by ELISA. Values are presented as the mean ± SD of at least eight individuals in each group. *, **, *P* < 0.05 and 0.01 vs. baseline or the phototherapy group, respectively (Student’s *t* test).

We next evaluated the impact of phototherapy on the expression profiles of total and OVA-specific immunoglobulins after the 15th OVA challenge. As shown in Fig. 2, OVA sensitization and the subsequent OVA challenge significantly induced the elevation of total and OVA-specific IgE and IgG1 levels, suggesting the activation of IgE- and Th2-mediated allergy responses in the FA group. Phototherapy suppressed the elevation of total and OVA-specific IgE and IgG1 levels but slightly induced total and OVA-specific IgG2a expression after OVA challenge, resulting in the amelioration of FA-like allergic diarrhea by daily phototherapy during OVA sensitization and the subsequent OVA challenge.

### Impact of phototherapy on T-cell phenotypes and FA-associated pro-Th2 cytokine expression in the intestine

**Fig. 3:**
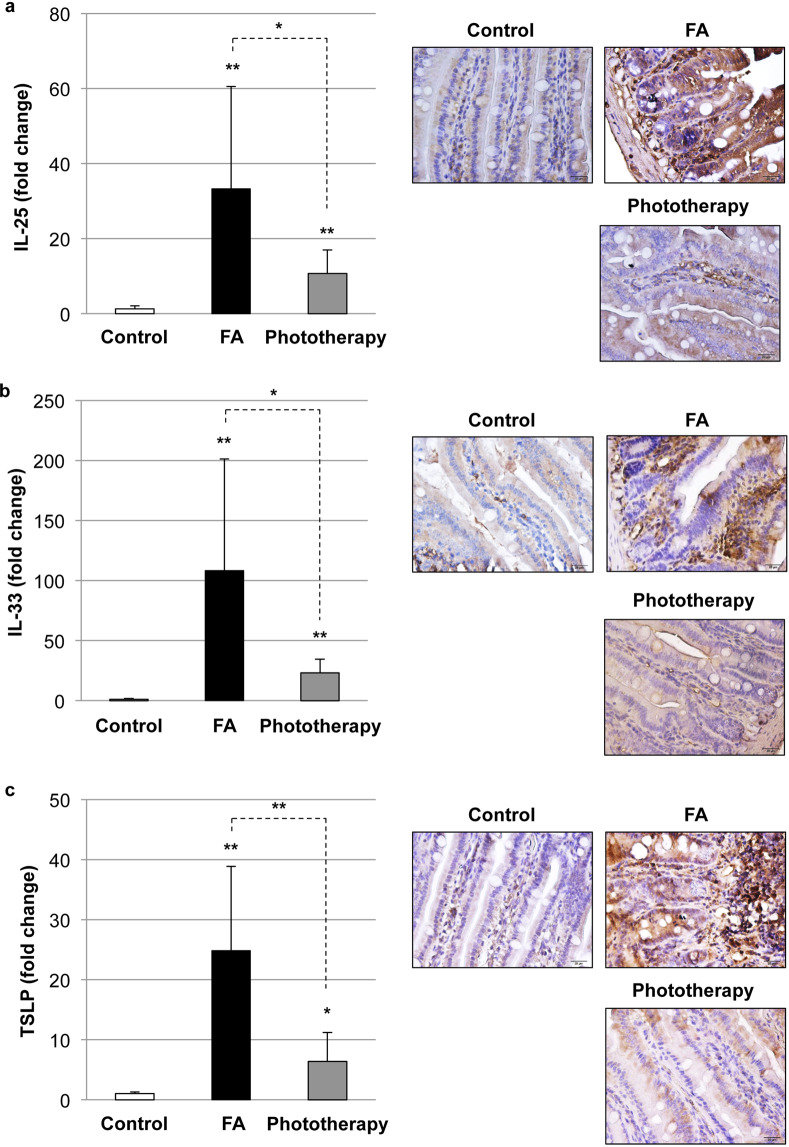
Impact of phototherapy on food allergy (FA)-associated pro-Th2 cytokine expression. Intestinal levels of IL-25 (**a**), IL-33 (**b**), and thymic stromal lymphopoietin (TSLP) (**c**) were evaluated by quantitative real-time PCR and immunohistochemistry (IHC). Left: values are presented as the mean ± SD of at least eight individuals in each group. *, **, *P* < 0.05 and 0.01 vs. the control or phototherapy group, respectively (Student’s *t* test). The pictures at ×400 magnification are representative of at least six individuals in each group. Scale bar: 20 μm.

We next explored the impact of phototherapy on T-cell phenotypes in the intestine. As shown in Supplementary Fig. 2, the expression levels of T-bet (Th1), GATA3 (Th2), RoRγt (Th17), and FoxP3 (regulatory T cells) were significantly elevated in the FA intestine, while phototherapy reduced their expression. Phototherapy may ameliorate the activation of both effector T cells and regulatory T cells during OVA-induced allergic diarrhea. We further explored the impact of phototherapy on the intestinal level of the pro-Th2 cytokines IL-25, IL-33, and thymic stromal lymphopoietin (TSLP), which are primarily produced by epithelial cells upon allergen challenge and associated with FA^36^. As shown in Fig. 3, intestinal levels of IL-25, IL-33, and TSLP were significantly increased in the FA group, while phototherapy suppressed their expression in the intestine.

### Impact of phototherapy on vitamin D_3_ metabolism in FA

**Fig. 4:**
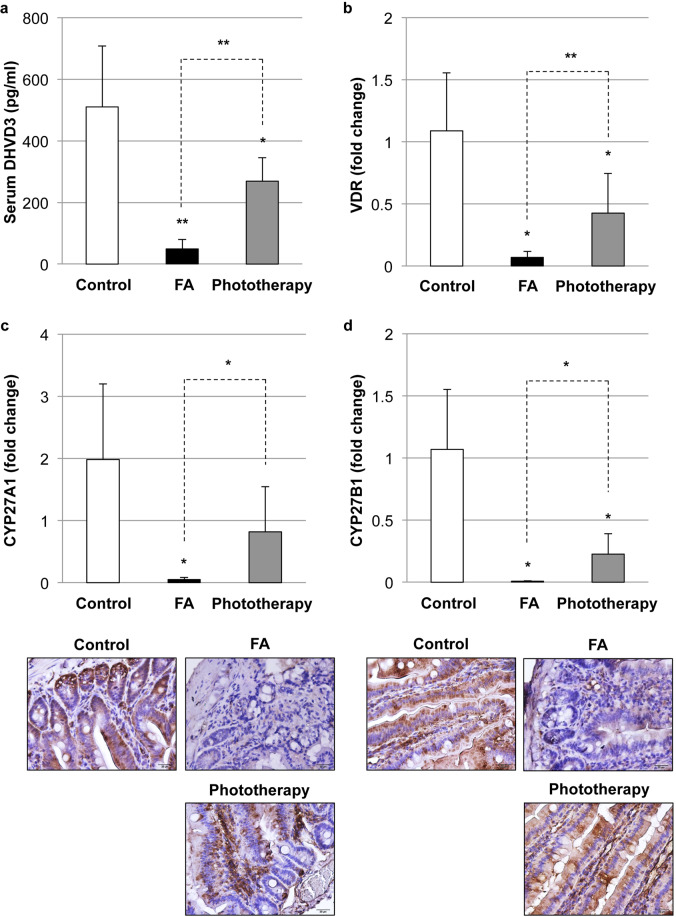
Impact of phototherapy on vitamin D_3_ metabolism in food allergy (FA). **a** Serum levels of vitamin D_3_ were measured by ELISA. Values are presented as the mean ± SD of at least eight individuals in each group. *, **, *P* < 0.05 and 0.01 vs. the control or phototherapy group, respectively (Student’s *t* test). Intestinal levels of vitamin D receptor (VDR) (**b**), sterol 27-hydroxylase (CYP27A1) (**c**), and 25-hydroxyvitamin D_3_-1α hydroxylase (CYP27B1) (**d**) were evaluated by quantitative real-time PCR and immunohistochemistry (IHC). Values are presented as the mean ± SD of at least six individuals in each group. *, **, *P* < 0.05 and 0.01 vs. the control or phototherapy group, respectively (Student’s *t* test). The pictures at ×400 magnification are representative of at least six individuals in each group. Scale bar: 20 μm.

To explore the impact of phototherapy on vitamin D_3_ status in FA, the serum level of the active form vitamin D_3_ (DHVD3) was quantified. As shown in Fig. 4a, vitamin D_3_ insufficiency was observed in the FA group. Corresponding to the reduction in serum vitamin D_3_, vitamin D receptor (VDR) expression in the intestine was also reduced in the FA group (Fig. 4b). On the other hand, phototherapy could induce the elevation of serum vitamin D_3_ in naïve mice (Supplementary Fig. 3a). Therefore, FA mice with phototherapy were able to ameliorate vitamin D_3_ insufficiency with higher expression of intestinal VDR than FA mice (Fig. 4). These data were negatively correlated with the expression profiles of total and OVA-specific IgE in FA with/without phototherapy (Fig. 2a).

We next explored the intestinal levels of the vitamin D-metabolizing enzymes sterol 27-hydroxylase (CYP27A1) and 25-hydroxyvitamin D_3_-1α hydroxylase (CYP27B1). As shown in Fig. 4c and d, the expression levels of CYP27A1 and CYP27B1 in the intestine were significantly reduced in the FA group compared with the control group. As shown in Supplementary Fig. 3b, phototherapy could induce the expression of intestinal CYP27A1 and CYP27B1 in naïve mice. Therefore, phototherapy could maintain these levels to some extent. These results suggest the involvement of intestinal vitamin D biosynthesis and a VDR-mediated mechanism of action of endogenous vitamin D_3_ in the amelioration of FA-like allergic diarrhea by phototherapy. The reduction in intestinal CYP27A1 and CYP27B1 levels may not be due to the sloughing of the epithelial lining from multiple episodes of allergic diarrhea in the FA group because of the similar expression profile of epithelial cell adhesion molecule, a marker for intestinal epithelial cells, in the control, FA, and phototherapy groups (Supplementary Fig. 3c). Taken together, the reduction in CYP27A1 and CYP27B1 is likely associated with FA-like allergic diarrhea.

### Impact of phototherapy on oxidative stress and antioxidant defense responses in FA

**Fig. 5:**
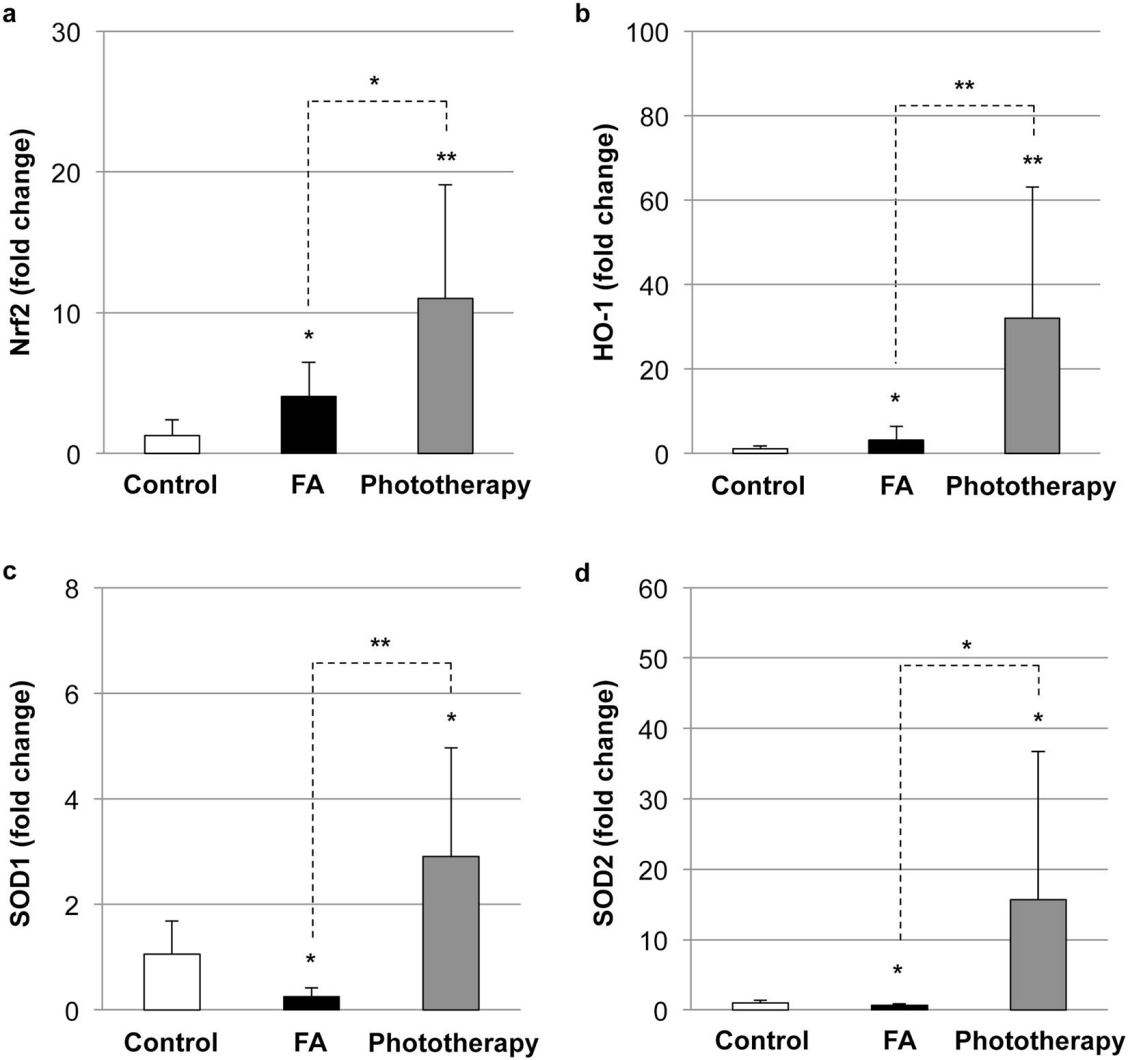
Impact of phototherapy on oxidative stress and antioxidant defense in food allergy (FA). Intestinal levels of nuclear factor erythroid 2-related factor 2 (Nrf2) (**a**), heme oxygenase-1 (HO-1) (**b**), superoxide dismutase 1 (SOD1) (**c**), and SOD2 (**d**) were evaluated by quantitative real-time PCR. Values are presented as the mean ± SD of at least eight individuals in each group. *, **, *P* < 0.05 and 0.01 vs. the control or phototherapy group, respectively (Student’s *t* test).

A recent study mentioned the association between FA and the oxidative stress response^37^. To further explore the impact of phototherapy on oxidative stress and antioxidant defense responses in FA, the intestinal levels of nuclear factor erythroid 2-related factor 2 (Nrf2), a master regulator of the antioxidant machinery, and antioxidant genes such as heme oxygenase-1 (HO-1), superoxide dismutase 1 (SOD1), and SOD2 were evaluated. As shown in Fig. 5a, intestinal Nrf2 expression was elevated in both the FA and phototherapy groups, while FA failed to induce SOD1 and SOD2 (Fig. 5c, d). Intriguingly, phototherapy activated intestinal Nrf2 expression in both naïve and FA mice (Supplementary Fig. 4) followed by the induction of antioxidant genes. Taken together, one of the potential mechanisms for the amelioration of FA by phototherapy may be the modulation of the oxidative stress response by Nrf2 activation.

### Impact of phototherapy on the fecal microbiota composition in FA

**Fig. 6:**
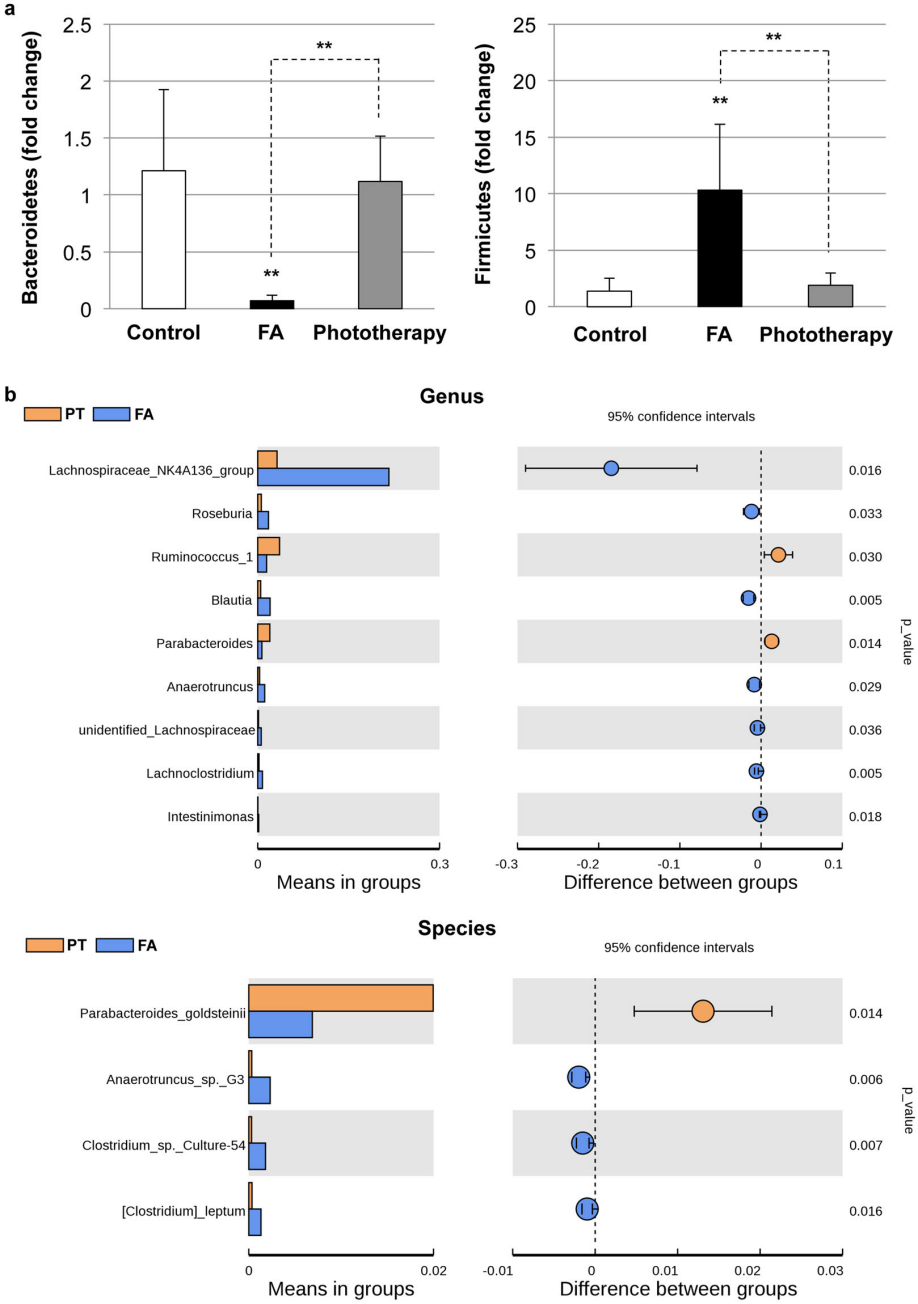
Altered fecal microbiota composition in food allergy (FA). **a** Quantitative real-time PCR for verification of the phyla *Bacteroidetes* and *Firmicutes*. Values are presented as the mean ± SD of at least eight individuals in each group. **P < 0.01 vs. the control or phototherapy group (Student’s *t* test). **b** Beta diversity comparison of gut microbiomes between the FA and phototherapy groups. The data indicate the abundance of species and the difference between groups (95% confidential interval) at the genus and species levels. FA food allergy, PT phototherapy.

We performed bacterial taxonomic analysis to investigate the impact of phototherapy on the fecal microbiota composition in FA. As shown in Supplementary Fig. 5a, FA induced a reduction in the phylum *Bacteroidetes* and an elevation in the phylum *Firmicutes*, which correspond to the ratio observed in clinical FA patients^38^. Quantitative real-time PCR analysis also demonstrated an elevation in the *Firmicutes*/*Bacteroidetes* ratio (196.9 ± 129.3, *P* < 0.0001 vs. control: 1.11 ± 0.547) in FA mice (Fig. 6a). In addition, phototherapy maintained a comparable *Firmicutes*/*Bacteroidetes* ratio (1.94 ± 1.71, *P* = 0.143) to the control, suggesting the impact of phototherapy on the maintenance of healthy microbiota. Linear discriminant analysis (LDA) effect size (LEfSe) analysis was then performed to discover biomarkers with significant differences among the three groups. As shown in Supplementary Fig. 5b, the genus *Lachnospiraceae_NK4A136_group* (phylum *Firmicutes*) and family *Peptococcaceae* (phylum *Firmicutes*) were enriched in FA mice. We next performed beta diversity comparison of the gut microbiomes between the FA and phototherapy groups at the genus and species levels. As shown in Fig. 6b, FA significantly increased the genus *Lachnospiraceae_NK4A136_group* (*P* = 0.016). On the other hand, the species *Parabacteroides_goldsteinii* (Phylum *Bacteroidetes*) was enriched in phototherapy-treated mice (*P* = 0.014).

### Application of FMT for functional evaluation of FA-associated dysbiosis

**Fig. 7:**
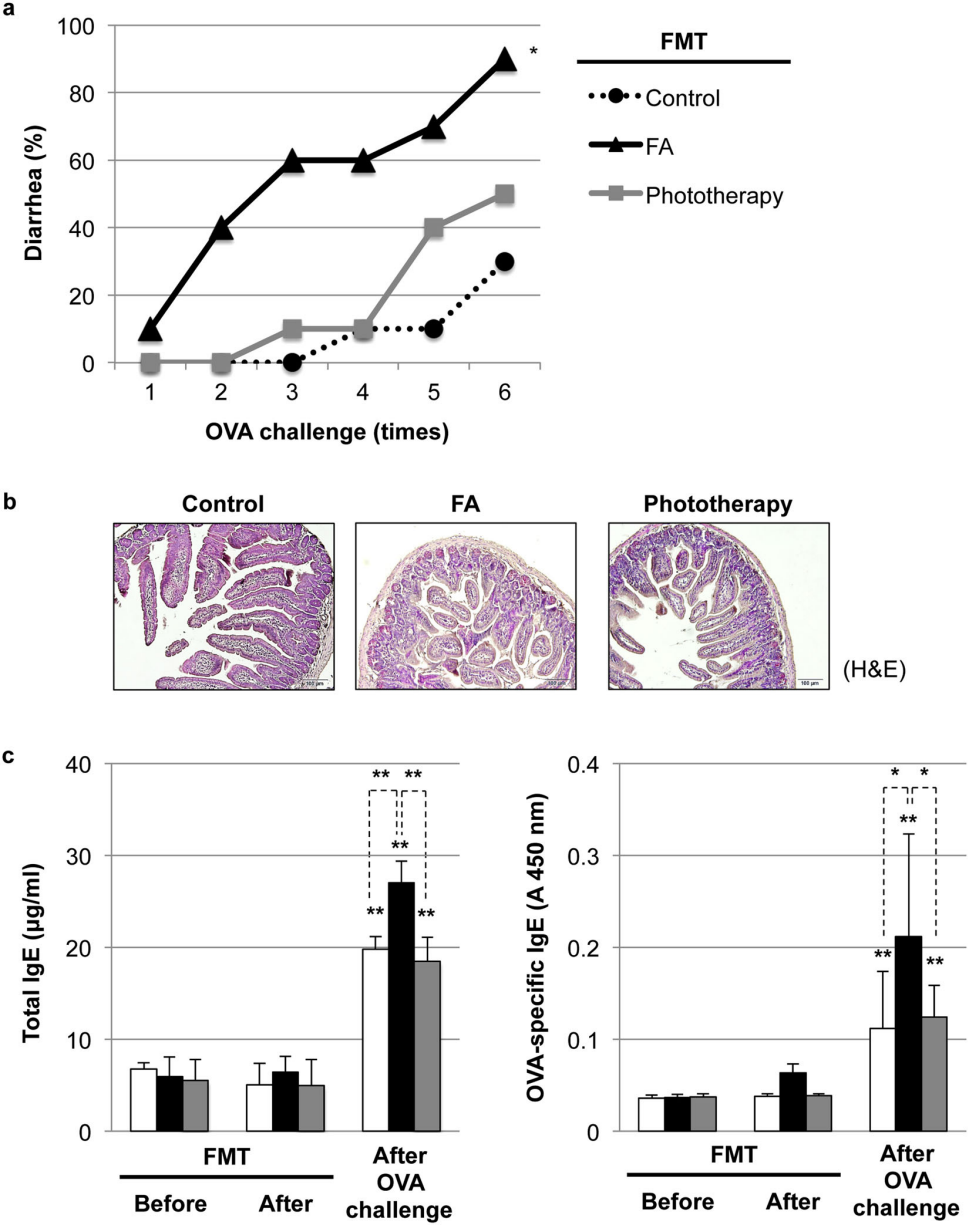
Application of fecal microbiota transplantation (FMT) for functional evaluation of food allergy (FA)-associated dysbiosis. **a** The incidence of allergic diarrhea in naïve mice with FMT. After antibiotic-induced dysbiosis, a 200-μl/day aliquot of the resuspended material (5-mg/ml feces from control, FA or phototherapy group, *n* = 10 per group) was given by oral gavage for 3 days. Two weeks after FMT, repeated intragastric (i.g.) OVA challenge was performed, and allergic diarrhea was assessed visually by monitoring mice for 30 min after each i.g. exposure. *P < 0.05 vs. the control-FMT group (Tukey’s multiple comparisons test). **b** Histological evaluation of the intestines of naïve mice with FMT after the 6th OVA challenge. The pictures at ×100 magnification are representative of ten individuals in each group. H&E hematoxylin and eosin staining. Scale bar: 100 μm. **c** Elevation of total and OVA-specific IgE after the 6th OVA challenge in FMT mice with donor FA feces (FA-FMT mice, black bar) as compared with the levels in control-FMT (white bar) or phototherapy-FMT (gray bar) mice. Values are presented as the mean ± SD of ten individuals in each group. *, **, *P* < 0.05 and 0.01 vs. baseline data before FMT or the indicated pair, respectively (Student’s *t* test).

We performed FMT to explore whether FA-associated dysbiosis directly transfers FA symptoms in naïve BALB/c mice. As shown in Supplementary Fig. 6a, antibiotics completely removed the original gut microbiota, and FMT with FA donor feces (FA-FMT) successfully introduced FA-associated dysbiosis in naïve BALB/c mice. On the other hand, FMT itself did not affect the expression profiles of circulating vitamin D_3_ (Supplementary Fig. 6b) and pro-Th2 cytokines (Supplementary Fig. 6c). As shown in Fig. 7a, allergic diarrhea occurred immediately after the 1st OVA challenge, and 90% (9/10) of FA-FMT mice experienced allergic diarrhea at the 6th OVA challenge. In addition, the incidence of allergic diarrhea in the phototherapy-FMT or control-FMT groups was 50% (5/10) and 30% (3/10), respectively, at the 6th OVA challenge. We confirmed the statistic significance between FA-FMT and control-FMT (*P* = 0.0164). Histological data revealed allergic inflammation and abnormal villous shortening by FA-FMT (Fig. 7b). Although FMT itself did not affect the levels of total and OVA-specific IgE, FA-FMT mice had higher levels of total and OVA-specific IgE after the 6th OVA challenge (Fig. 7c). This evidence suggests the existence of unique microbacteria in FA donor feces for the induction of the OVA-specific IgE response and IgE-mediated allergic diarrhea. We also confirmed vitamin D_3_ insufficiency in all FMT groups (Supplementary Fig. 6b) due to the induction of dysbiosis by OVA challenge (data not shown). However, the expression levels of vitamin D_3_, CYP27A1, and CYP27B1 in control-FMT and phototherapy-FMT were relatively higher than those in FA-FMT (Supplementary Fig. 6b). Although OVA challenge induced the elevation of intestinal IL-25, IL-33, and TSLP in all FMT groups, the control-FMT and phototherapy-FMT groups had relatively lower expression of IL-33 and TSLP than FA-FMT (Supplementary Fig. 6c). We also confirmed the relatively higher expression of Nrf2 by phototherapy-FMT treatment (Supplementary Fig. 7a), resulting in a lower incidence of allergic diarrhea.

### Application of FMT for therapeutic modulation of diseased microbiota

**Fig. 8:**
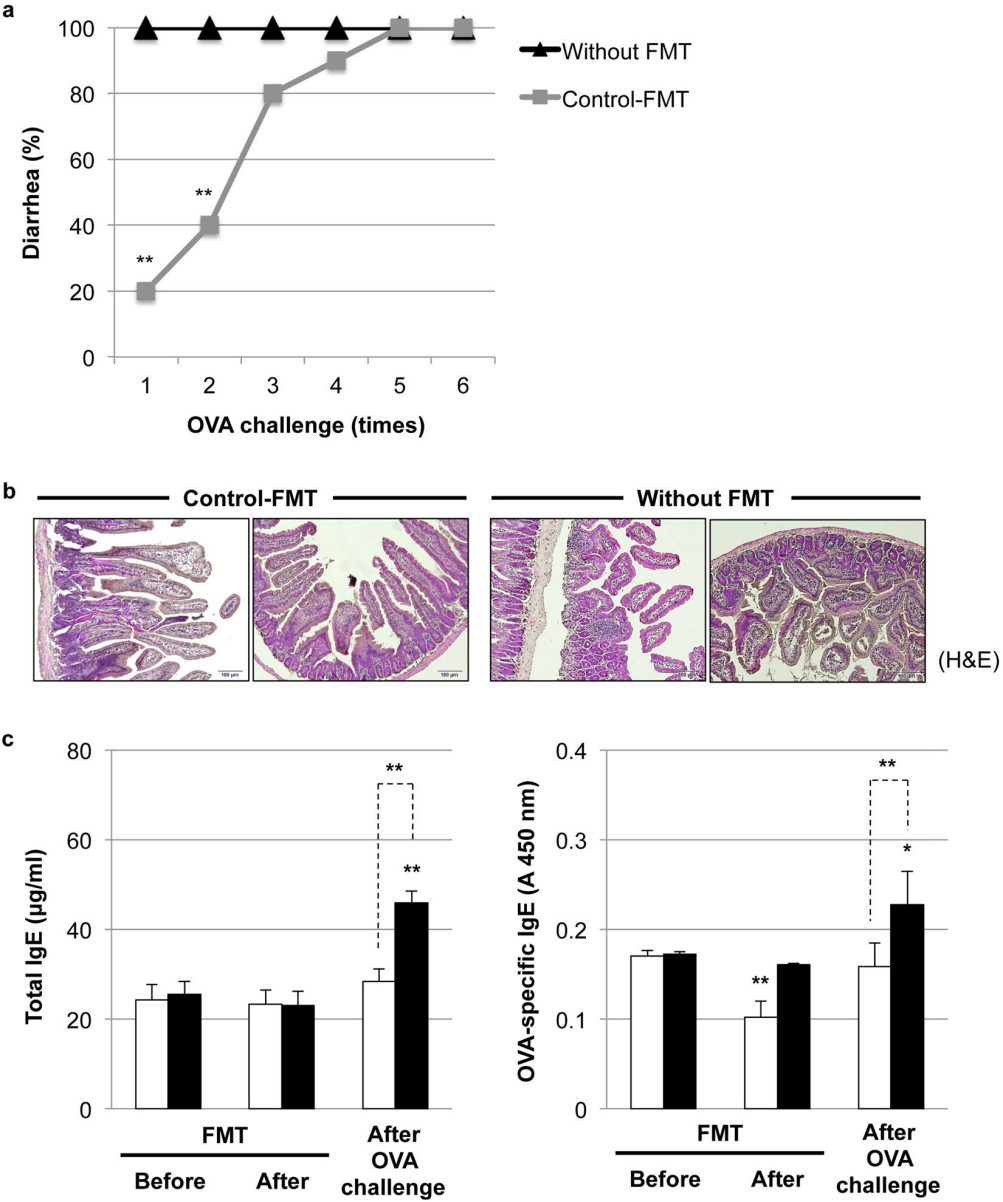
Application of fecal microbiota transplantation (FMT) for the therapeutic modulation of diseased microbiota. **a** Therapeutic modulation of gut microbiota by FMT in FA mice. After the development of FA and following antibiotic-induced dysbiosis, FMT with control donor feces (control-FMT) was administered to FA mice (*n* = 10). FA mice without FMT (*n* = 5) were set as a comparison. Allergic diarrhea was assessed visually by monitoring mice for 30 min after each i.g. exposure. **P < 0.01 vs. FA mice without FMT (Tukey’s multiple comparisons test). **b** Histological evaluation of the intestines of FA mice with/without control-FMT after the 6th OVA challenge. The pictures at ×100 magnification are representative of ten individuals in each group. H&E hematoxylin and eosin staining. Scale bar: 100 μm. **c** Reduced expression of total and OVA-specific IgE after the 6th OVA challenge in FA mice with control-FMT (white bar). Black bar: FA mice without FMT. Values are presented as the mean ± SD of ten individuals in each group. *, **, *P* < 0.05 and 0.01 vs. FA mice before FMT or the indicated pair, respectively (Student’s *t* test).

To examine the impact of gut microbiota on FA from another perspective, we performed control-FMT to FA mice. As expected, control-FMT improved FA-associated dysbiosis by modulating the *Firmicutes*/*Bacteroidetes* ratio (0.726 ± 0.405) (Supplementary Fig. 8a). Similar to the previous observation (Supplementary Fig. 6b), FMT itself did not recover vitamin D_3_ insufficiency (Supplementary Fig. 8b) and maintained intestinal pro-Th2 cytokine expression in FA mice (Supplementary Fig. 8c). As shown in Fig. 8a, control-FMT mice showed delayed onset of allergic diarrhea. Although allergic diarrhea was observed at the 6th OVA challenge in control-FMT mice, histological data revealed a better intestinal morphology in FA mice receiving control-FMT (Fig. 8b). We also confirmed the reduced expression of total and OVA-specific IgE by control-FMT (Fig. 8c). The replacement of diseased microbiota with healthy microbiota by control-FMT reduced the elevation of intestinal IL-25, IL-33, and TSLP but enhanced Nrf2 expression (Supplementary Fig. 7b). This evidence suggests the therapeutic potential of FMT for FA-like allergic diarrhea partly through the modulation of gut microbiota.

## Discussion

The importance of sunlight exposure on mental and physical conditions has been well recognized since time immemorial. However, promoting the avoidance of sunlight exposure in recent years has led to the risk of not receiving beneficial effects such as vitamin D_3_ biogenesis. In this study, we used full spectrum light, which may reduce the risk of excessive UV exposure, as a light source for phototherapy and demonstrated its beneficial impact on FA-like allergic diarrhea (Figs. 1–3).

Although the mode of action of phototherapy was not fully clarified in this study, one of the potential mechanisms underlining the beneficial impact of phototherapy should be the improvement in FA-associated vitamin D_3_ insufficiency. FA mice showed a reduction in intestinal VDR, CYP27A1, and CYP27B1 expression, resulting in the impairment of vitamin D biosynthesis and VDR-mediated mechanism of action of endogenous vitamin D_3_. On the other hand, phototherapy could prevent the FA-associated reduction in systemic vitamin D_3_ and intestinal genes for vitamin D_3_ metabolism (Fig. 4). OVA-sensitized *Cyp27b1*-knockout mice had an increase in total and OVA-specific IgE concentrations^39^, suggesting the regulation of the IgE-mediated allergic response by endogenous vitamin D_3_. Vitamin D_3_-mediated activation of VDR and the subsequent induction of Nrf2-antioxidant signaling (Fig. 5) may play an important role in maintaining the integrity of the intestinal mucosal barrier^40^, resulting in the amelioration of allergic diarrhea.

Another potential mechanism of phototherapy in the prevention of FA could be the modulation of gut microbiota (Fig. 6). In line with our current data, a recent study demonstrated the existence of a link between vitamin D_3_ deficiency and altered gut microbiota composition characterized by the reduction in the phylum *Bacteroidetes* and the elevation of the phylum *Firmicutes*^41^. Interestingly, FA-associated dysbiosis could directly transfer FA symptoms in naïve BALB/c mice (Fig. 7), suggesting the existence of unique microbacteria in FA donor feces that can induce the OVA-specific IgE response and subsequent allergic diarrhea. In this study, the genus *Lachnospiraceae_NK4A136_group* was identified as an FA-associated microbacteria. In concordance with our study, another study also mentioned the elevation of the *Lachnospiraceae_NK4A136_group* in patients with wheat-dependent exercise-induced anaphylaxis^42^. The replacement of FA-associated bacteria with healthy microbiota may be a promising strategy for FA therapeutics (Fig. 8). In addition, one of the beneficial bacterial species, *Parabacteroides goldsteinii* (phylum *Bacteroidetes*), was enriched in phototherapy-treated mice. Interestingly, *Parabacteroides goldsteinii* was enriched in the offspring of goat-milk-fed pregnant mice and may provide a protective effect against atopy development and alleviate allergen-induced airway inflammation in offspring^43^. Further studies are necessary to understand the impact of these unique microbes on FA development and prevention.

There are several limitations in this study. First, our animal model^44^ is recognized as a model for FAs but this model does not completely mimic FA symptoms such as IgE-mediated anaphylaxis^45^. We need to carefully evaluate the impact of phototherapy on other FA symptoms as well as adverse events caused by phototherapy before we proceed to clinical application. Second, the correlation between vitamin D_3_ and microbiota composition was not fully clarified in this study. Although we have no data to reveal the direct impact of vitamin D_3_ supplementation on gut microbiota composition, the replacement of gut microbiota by FMT could not alter the vitamin D_3_ level (Supplementary Figs. 6 and 8). Vitamin D_3_ and gut microbiota composition may be independent of each other. Vitamin D_3_ may be important for gut homeostasis, but its expression may not be regulated by the gut microbiota composition.

In summary, we proposed phototherapy as an alternative approach for the prevention and treatment of FA-like allergic diarrhea, and it may maintain circulating vitamin D_3_ and healthy microbiota for the prevention of FA. There are several clinical trials exploring the effect of vitamin D supplementation in the prevention of FAs, while there is a lack of consistent data addressing the preventive ability of vitamin D supplementation for FAs^46^. Of note, a recent randomized controlled trial revealed that daily high-dose vitamin D supplementation (30 μg, 1200 IU) does not prevent allergic sensitization but rather increases the risk of milk allergy in infants^47^. How to find an optimal dose of vitamin D_3_ for effective prevention of FAs is one of the important issues for reducing the risk of adverse effects. Although it is too early to make a conclusion, a better approach to the prevention of FAs could be the use of phototherapy due to the induction of endogenous vitamin D_3_ production at an optimal concentration as well as the modulation of gut microbiota. The exposure time and duration of phototherapy should be further considered for future clinical applications.

## Methods

### Mice

Female BALB/c mice, 4 weeks of age, were obtained from the National Laboratory Animal Breeding and Research Center (Taipei, Taiwan). All animals were maintained in specific pathogen-free animal facilities with water and commercial rodent food provided ad libitum. Our experimental design was reviewed and approved by the Institutional Animal Care and Use Committee (Approval No. 2018122119) of Kaohsiung Chang Gung Memorial Hospital.

### Establishment of a FA model and phototherapy

We prepared OVA-induced FA mice according to a previously described protocol^44^ with minor modifications (Supplementary Fig. 9a). Briefly, BALB/c mice (*n* = 26) were divided into two groups: the FA group (*n* = 14) and the phototherapy group (*n* = 12). The phototherapy group received daily exposure to full spectrum light (color temperature 5500 K, color rendition indexes >90; Chang Gung Biotechnology, Taipei, Taiwan) (12 h/day) throughout the entire experiment (9 weeks). The distance between the animals and the full spectrum light was kept at ~45 cm (Supplementary Fig. 9b). Both groups were intraperitoneally immunized twice with a precipitate (100 μl/mouse) of OVA (60 μg, Sigma-Aldrich, St Louis, MO, USA) and aluminum hydroxide (1 mg, Thermo Fisher Scientific Inc., Rockford, IL, USA). After the second immunization with a 2-week interval, repeated intragastric (i.g.) OVA challenge was performed 15 times (every 2–3 days, 15 mg in a final volume of 150 μl of PBS). Naïve mice (*n* = 8) without OVA immunization were treated with PBS as a control. Allergic diarrhea was assessed visually by monitoring mice for 30 min after each i.g. exposure. Experimental mice were sacrificed after the final OVA challenge, and intestinal tissues, blood, and stool samples were collected for histological and biochemical analyses.

### Histological assessment of FA

Experimental mice were sacrificed after the final OVA challenge, and the intestinal specimens were embedded in paraffin. The paraffin sections (5 μm) were dewaxed and stained with H&E or toluidine blue for evaluation of inflammation and mast cell infiltration. At least six randomly chosen high-power fields (HPFs) of each section were examined to yield the mean number of mast cells per HPF in the intestine.

### Immunohistochemistry

After peroxidase blocking, antigen retrieval and blocking, the slides were incubated with rat monoclonal antibody against Mcpt-1 (200× dilution; Thermo Fisher Scientific Inc.), mouse monoclonal antibody against IL-25 (500× dilution; Thermo Fisher Scientific Inc.), mouse monoclonal antibody against IL-33 (200× dilution; Thermo Fisher Scientific Inc.), rabbit polyclonal antibody against TSLP (400× dilution; Thermo Fisher Scientific Inc.), rabbit polyclonal antibody against CYP27A1 (500× dilution; Thermo Fisher Scientific Inc.), or rabbit polyclonal antibody against CYP27B1 (500× dilution; Thermo Fisher Scientific Inc.) overnight at 4 °C followed by incubation with Rat or Mouse/Rabbit Probe HRP (BioTnA, Kaohsiung, Taiwan) for 30 min. After washing, chromogen development was performed by 3,3′-diaminobenzidine staining. Counter staining was carried out using hematoxylin. The slides were rinsed with H_2_O and covered with resin-based mounting medium (BioTnA) after dehydration.

### Total and OVA-specific immunoglobulins

The serum levels of total and OVA-specific IgG1, IgG2a, and IgE were determined by ELISA^48^. For evaluation of total immunoglobulin levels, anti-mouse IgG1, IgG2a, or IgE (250× dilution; BD Biosciences, San Jose, CA, USA) was coated onto 96-well microplates (Nalgene Nunc International, Roskilde, Denmark) and incubated overnight at 4 °C. For evaluation of OVA-specific immunoglobulin levels, OVA solution (50 mg/ml) was coated instead. After blocking, serum samples with appropriate dilution (total IgG1; 10,000× dilution, total IgG2a; 2000× dilution, total IgE; 250× dilution, OVA-specific IgG1; 2,000,000× dilution, OVA-specific IgG2a; 200,000× dilution, OVA-specific IgE; 10× dilution) were added to each well and incubated for 2 h at 37 °C. After washing, biotin-conjugated anti-mouse IgG1, IgG2a, or IgE (250× dilution; BD Biosciences) were added and incubated for 1.5 h at 37 °C followed by incubation with streptavidin-HRP (200× dilution; R&D Systems, Minneapolis, MN, USA) for 1 h. After washing, 1-Step Ultra TMB Substrate Solution (Thermo Fisher Scientific Inc.) was added for color development. After the addition of stop solution, the absorbance at 450 nm was measured using a Victor X4 Multilabel Plate Reader (PerkinElmer, Waltham, MA, USA).

### RNA extraction and quantitative real-time PCR analysis

RNA was extracted from the mouse intestine using TRIzol (Thermo Fisher Scientific Inc.) and reverse transcribed with a High-Capacity cDNA Reverse Transcription Kit (Thermo Fisher Scientific Inc.). The expression levels of IL-25, IL-33, TSLP, VDR, CYP27A1, CYP27B1, Nrf2, HO-1, SOD1, and SOD2 were quantified using quantitative real-time PCR (Applied Biosystems, Foster, CA, USA) with specific primer sets as shown in Supplementary Table 1. β-Actin was used as an internal control for normalization.

### Vitamin D_3_ ELISA

The serum level of the active form of vitamin D_3_ was measured using a General 1,25-Dihydroxyvitamin D_3_ (DHVD3) ELISA Kit (MyBioSource, Inc., San Diego, CA, USA). Briefly, 50 μl of standard and serum samples (undiluted) were added to pre-coated plate with DHVD3 monoclonal antibody, and biotin-labeled DHVD3 analogs were immediately added for the competitive inhibition reaction. After incubation for 1 h at 37 °C, unbound conjugate was washed off followed by incubation with avidin-conjugated HRP (100 μl) for 30 min at 37 °C. After washing and addition of substrate solution (90 μl) for 15 min at 37 °C, stop solution (50 μl) was added and the absorbance at 450 nm was determined using a Victor X4 Multilabel Plate Reader (PerkinElmer).

### Stool sample preparation, 16S ribosomal RNA gene amplicon sequencing, and fecal microbiota analysis

Stool samples were snap-frozen in liquid nitrogen before storage at −80 °C. Fecal DNA isolation, 16S rRNA gene amplicon sequencing and data analysis were performed by BIOTOOLS Co., Ltd., (New Taipei, Taiwan). Briefly, total genomic DNA was extracted using the CTAB/SDS method, and DNA was diluted to 1 ng/μl using sterile water. For 16S rRNA sequencing, the V4 region was amplified with a specific primer (515F-806R). All PCRs were carried out with Phusion® High-Fidelity PCR Master Mix (New England Biolabs, Ipswitch, MA, USA). The PCR products were monitored on a 2% agarose gel, and samples with bright main strips between 400 and 450 bp were chosen and purified with a Qiagen Gel Extraction Kit (Qiagen, Hilden, Germany). Sequencing libraries were generated using the NEBNext® Ultra™ DNA Library Pre Kit for Illumina® (New England Biolabs) following the manufacturer’s recommendations, and index codes were added. The library was sequenced on an Illumina platform (Illumina, San Diego, CA, USA), and 250-bp paired-end reads were generated. Paired-end reads were assigned to samples based on their unique barcode and truncated by cutting off the barcode and primer sequence. Paired-end reads were merged using FLASH (V1.2.7, http://ccb.jhu.edu/software/FLASH/), and the splicing sequences were called raw tags. Quality filtering of the raw tags was performed under specific filtering conditions to obtain the high-quality clean tags according to the QIIME quality control process (V1.7.0, http://qiime.org/index.html). The tags were compared with the reference database (Gold database, http://drive5.com/uchime/uchime_download.html) using the UCHIME algorithm (http://www.drive5.com/usearch/manual/uchime_algo.html) to detect chimera sequences (http://www.drive5.com/usearch/manual/chimera_formation.html). Then, the chimera sequences were removed to obtain effective tags.

For fecal microbiota analysis, alpha and beta diversity were determined based on output normalized data with QIIME software (V1.7.0). LEfSe analysis was performed to determine most abundant taxa among groups. A histogram of LDA scores is shown as the result of LEfSe analysis. LDA scores larger than 4 were displayed in the final output. A *t*-test was performed to determine species with significant variation between groups at various taxon ranks, including phylum, class, order, family, genus, and species.

### Quantitative real-time PCR analysis for bacterial DNA

Fecal bacterial DNA was extracted from the stool using a QIAamp Fast DNA Stool Mini Kit (Qiagen), and the expression levels of *Bacteroidetes* and *Firmicutes* were quantified using quantitative real-time PCR (Applied Biosystems) with specific primer sets as shown in Supplementary Table 2^49^. Total bacteria were used as an endogenous control to normalize gene expression.

### FMT

To explore the impact of FA-associated dysbiosis on FA symptoms, FMT was conducted on naïve mice. Before conducting FMT, naïve BALB/c mice (*n* = 30) received therapeutic-dose ampicillin (500 mg/kg) by oral gavage for 14 consecutive days^50^ with ampicillin (1 g/l) in drinking water ad libitum^51^ for antibiotic-induced gut dysbiosis. Then, a 200-μl/day aliquot of the resuspended material (5-mg/ml feces from the control, FA or phototherapy group, *n* = 10 per group) was given by oral gavage for 3 days^51^. To explore the therapeutic potential of FMT for the modulation of FA-associated dysbiosis, established FA mice (*n* = 10) received antibiotic-induced gut dysbiosis followed by FMT with donor control feces. The microbiota composition before and after FMT was evaluated by quantitative real-time PCR analysis. Two weeks after FMT, OVA challenge was performed and FA symptoms were evaluated as described above.

### Statistical analysis

Unpaired two-tailed Student’s *t* test was used to determine the significance of the difference between normally distributed means of the expression ratio in two groups. Tukey’s multiple comparisons test was used to determine the significance of the difference between each pair of means with appropriate adjustment for multiple testing. Each sample was tested in triplicate, and the results are indicated as the mean ± SD. Statistical analysis was performed using SPSS software version 22 (IBM, Armonk, NY, USA) or GraphPad Prism 7 software (GraphPad Software, La Jolla, CA, USA). A value of *P* < 0.05 was considered to be significant.

### Reporting summary

Further information on research design is available in the Nature Research Reporting Summary linked to this article.

## Supplementary information

Supplementary Information

Reporting Summary

## Data Availability

Raw data from the fecal microbiota analysis are deposited in the SRA database public repository from NCBI within the Bioproject accession number PRJNA690991.
