# Supplementary material for: Daily full spectrum light exposure prevents food allergy-like allergic diarrhea by modulating vitamin D3 and microbiota composition
Source: NPJ Biofilms Microbiomes. 2021 May 6;7:41. doi: 10.1038/s41522-021-00213-8 (PMC8102508; doi:10.1038/s41522-021-00213-8)
Supplement: Supplementary file 1 — Supplementary Information [file 41522_2021_213_MOESM1_ESM.pdf]

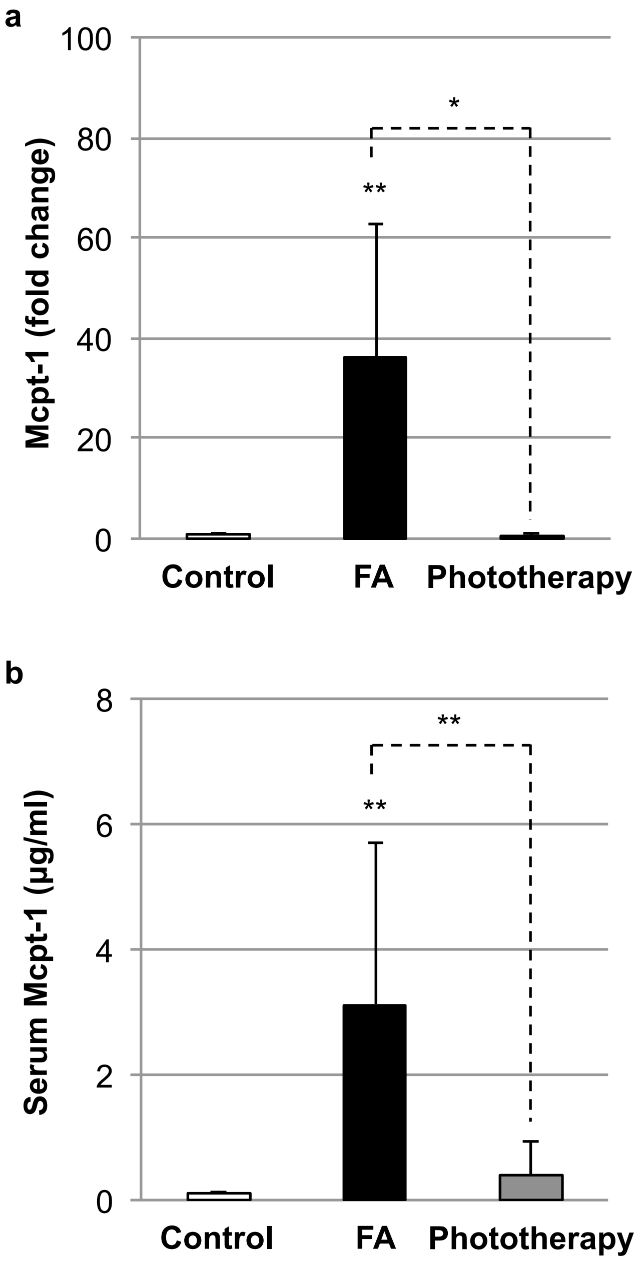

Supplementary Figure 1. The elevation of mast cell protease 1 (Mcpt-1) in food allergy (FA). FA-associated mucosal mast cell activation in the intestine (a) and the circulating level of Mcpt-1 (b) were evaluated by quantitative real-time PCR and ELISA, respectively. Values are presented as the mean ± SD of at least eight individuals in each group. *, **, $P< 0.05$ and 0.01 *vs.* FA mice, respectively (Student's *t*-test).

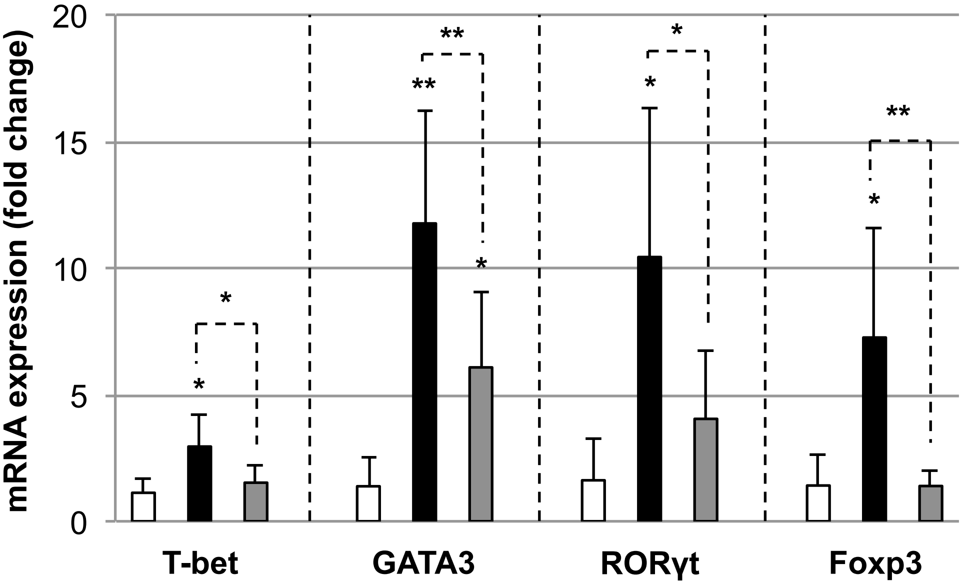

Supplementary Figure 2. Impact of phototherapy on T cell phenotypes. Intestinal levels of T-bet (Th1), GATA3 (Th2), RoRγt (Th17) and Foxp3 (regulatory T cells, Treg) were determined by quantitative real-time PCR analysis. Values are presented as the mean ± SD of at least eight individuals in each group. White bar: control, black bar: FA, gray bar: phototherapy. *, **, *P*< 0.05 and 0.01 *vs.* the control group, respectively (Student's *t*-test).

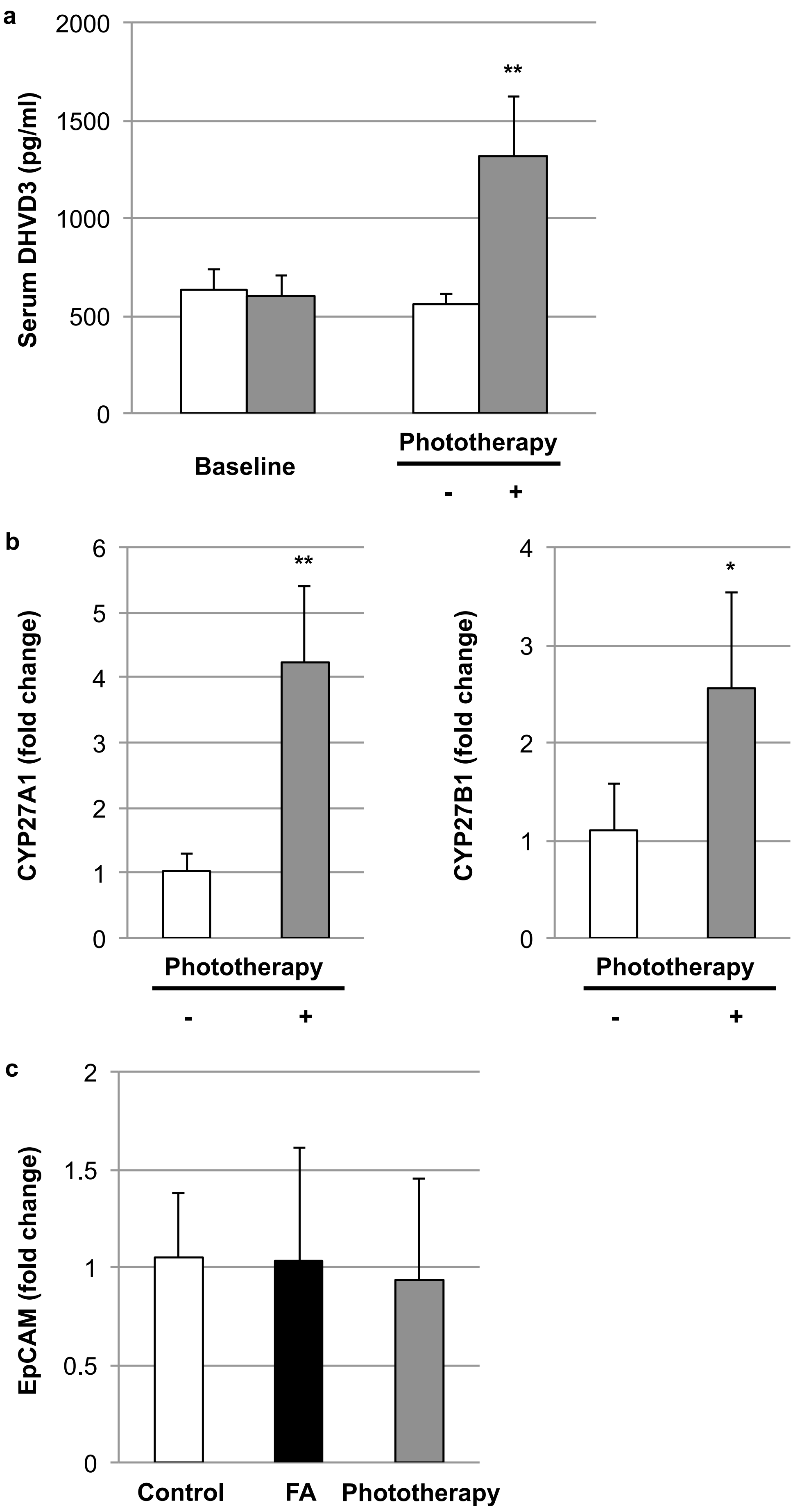
a
Serum DHVD3 (pg/ml)
2000
1500
1000
500
0
**
Baseline
Phototherapy
-
+
b
CYP27A1 (fold change)
6
5
4
3
2
1
0
**
Phototherapy
-
+
CYP27B1 (fold change)
4
3
2
1
0
*
Phototherapy
-
+
c
EpCAM (fold change)
2
1.5
1
0.5
0
Control
FA
Phototherapy

Supplementary Figure 3. Impact of phototherapy on vitamin D_3 metabolism in naïve mice. (a) Serum levels of vitamin D_3 were measured by ELISA. Values are presented as the mean ± SD of five individuals in each group. **, *P*< 0.01 (Student's *t*-test) *vs.* the control group. (b) Intestinal levels of sterol 27-hydroxylase (CYP27A1) and 25-hydroxyvitamin D_3-1α hydroxylase (CYP27B1) were evaluated by quantitative real-time PCR. Values are presented as the mean ± SD of five individuals in each group. *, **, *P*< 0.05 and 0.01 *vs.* the control group, respectively (Student's *t*-test). (c) The expression profiles of epithelial cell adhesion molecule (EpCAM) in the control, food allergy (FA) and phototherapy groups were evaluated by quantitative real-time PCR.

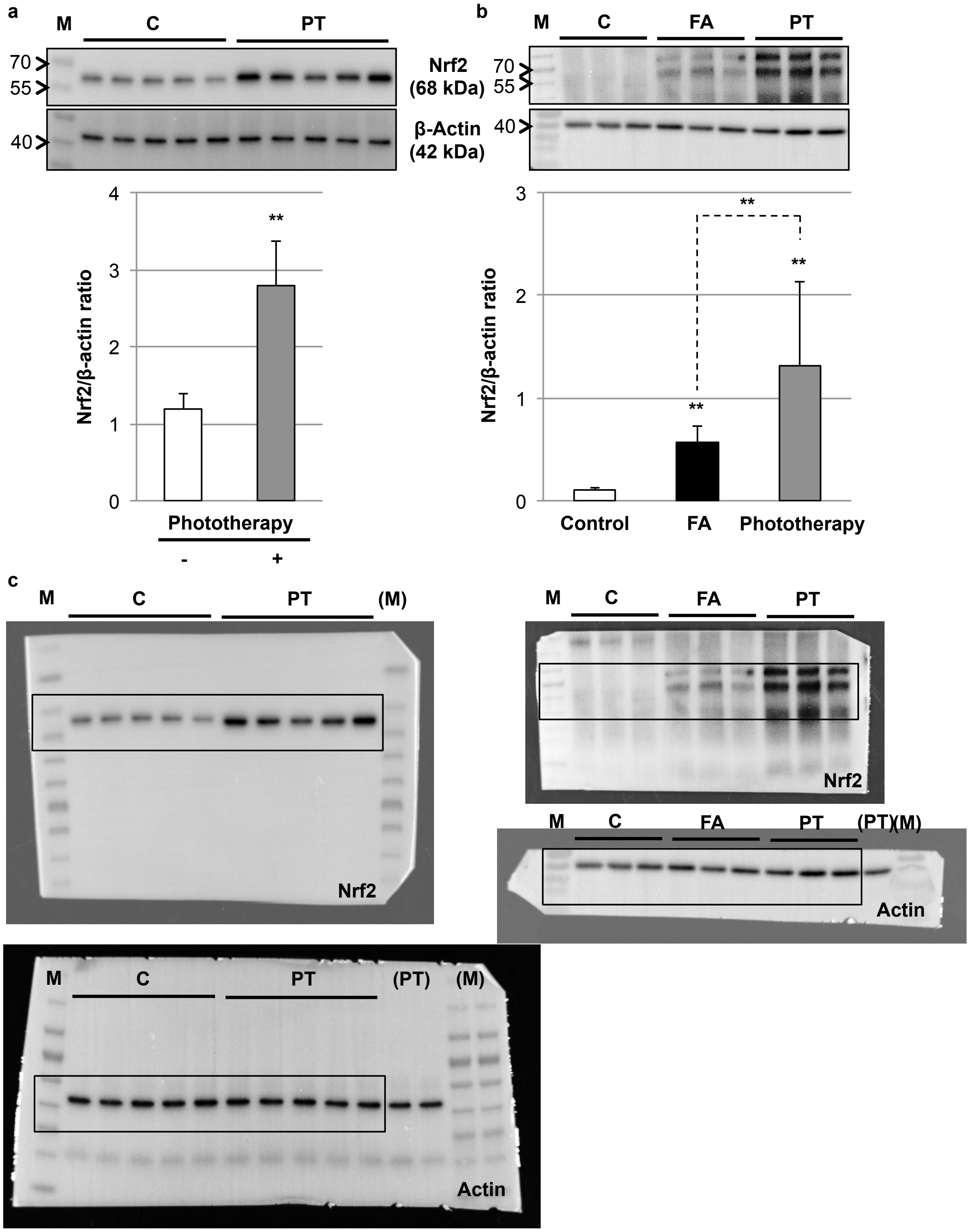

a
M
C
PT
70
55
40
Nrf2
(68 kDa)
β-Actin
(42 kDa)
Nrf2/β-actin ratio
4
3
2
1
0
**
Phototherapy
-
+
b
M
C
FA
PT
70
55
40
Nrf2/β-actin ratio
3
2
1
0
**
**
**
Control
FA
Phototherapy
c
M
C
PT
(M)
Nrf2
M
C
FA
PT
Nrf2
M
C
FA
PT
(PT)
(M)
Actin
M
C
PT
(PT)
(M)
Actin

Supplementary Figure 4. Impact of phototherapy on intestinal nuclear factor erythroid 2-related factor 2 (Nrf2) expression in naïve and food allergy (FA) mice. (a) Phototherapy for 9 weeks significantly enhanced the intestinal Nrf2 expression. Values are presented as the mean ± SD of five individuals in each group. **, $P< 0.01$ *vs.* the control group (Student's *t*-test). (b) FA induced intestinal Nrf2 expression, and its expression was further enhanced by phototherapy. The blot data are representative of three independent experiments. They are derived from the same experiment and processed in parallel. M: molecular weight marker, C: control, FA: food allergy, PT: phototherapy. Values are presented as the mean ± SD of three individuals in each group. **, $P< 0.01$ *vs.* the control or phototherapy group (Student's *t*-test). (c) Full, un-cropped images of all blots. Some blot data include additional samples or molecular weight markers, which are excluded in the cropped image (a and b).

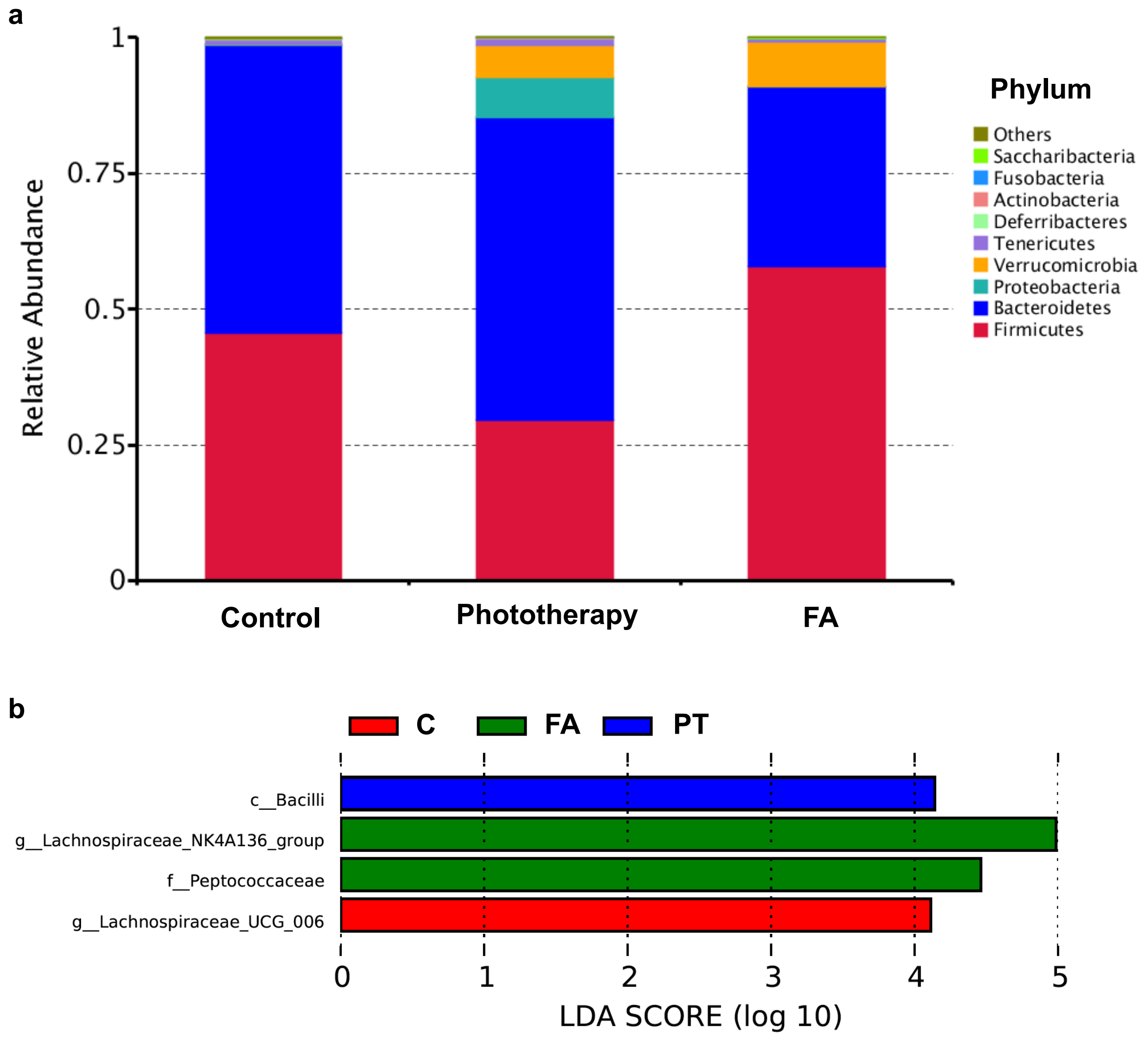

Supplementary Figure 5. Fecal microbiota composition. (a) Bacterial phyla in the control, FA and phototherapy groups (n=3 in each group). (b) Linear discriminant analysis (LDA) effect size (LEfSe) analysis was performed to determine the most abundant taxa among groups. Only genera with LDA scores greater than 4 are displayed. C: control, FA: food allergy, PT: phototherapy.

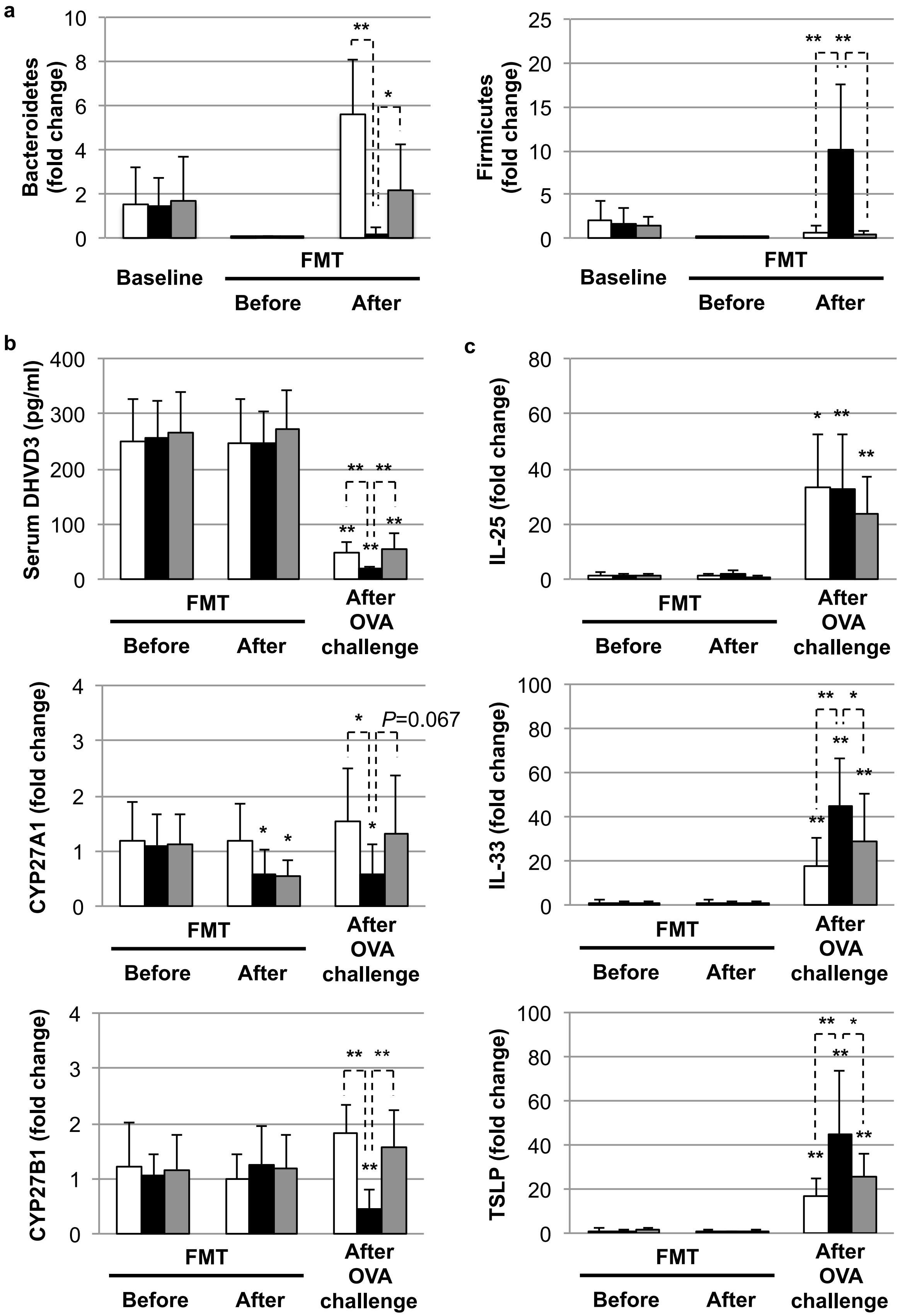
a
Bacteroidetes (fold change)
Baseline
FMT
Before
After
Firmicutes (fold change)
b
Serum DHVD3 (pg/ml)
After OVA challenge
CYP27A1 (fold change)
P=0.067
CYP27B1 (fold change)
c
IL-25 (fold change)
IL-33 (fold change)
TSLP (fold change)

Supplementary Figure 6. Impact of fecal microbiota transplantation (FMT) on the fecal microbiota composition, vitamin D_3 metabolism and intestinal cytokine expression in naïve mice. (a) Naïve BALB/c mice received FMT with control donor feces (control-FMT, white bar, n=10), food allergy (FA) donor feces (FA-FMT, black bar, n=10) or phototherapy donor feces (phototherapy-FMT, gray bar, n=10). The phyla *Bacteroidetes* and *Firmicutes* were evaluated by quantitative real-time PCR. *, **, $P< 0.05$ and 0.01, respectively (Student's *t*-test). (b) Serum levels of vitamin D_3 or intestinal levels of sterol 27-hydroxylase (CYP27A1) and 25-hydroxyvitamin D_3-1α hydroxylase (CYP27B1) were evaluated by ELISA and quantitative real-time PCR, respectively. *, **, $P< 0.05$ and 0.01 *vs.* baseline or the indicated pair, respectively (Student's *t*-test). (c) Intestinal levels of IL-25, IL-33 and thymic stromal lymphopoietin (TSLP) were evaluated by quantitative real-time PCR. *, **, $P< 0.05$ and 0.01 *vs.* baseline or the indicated pair, respectively (Student's *t*-test).

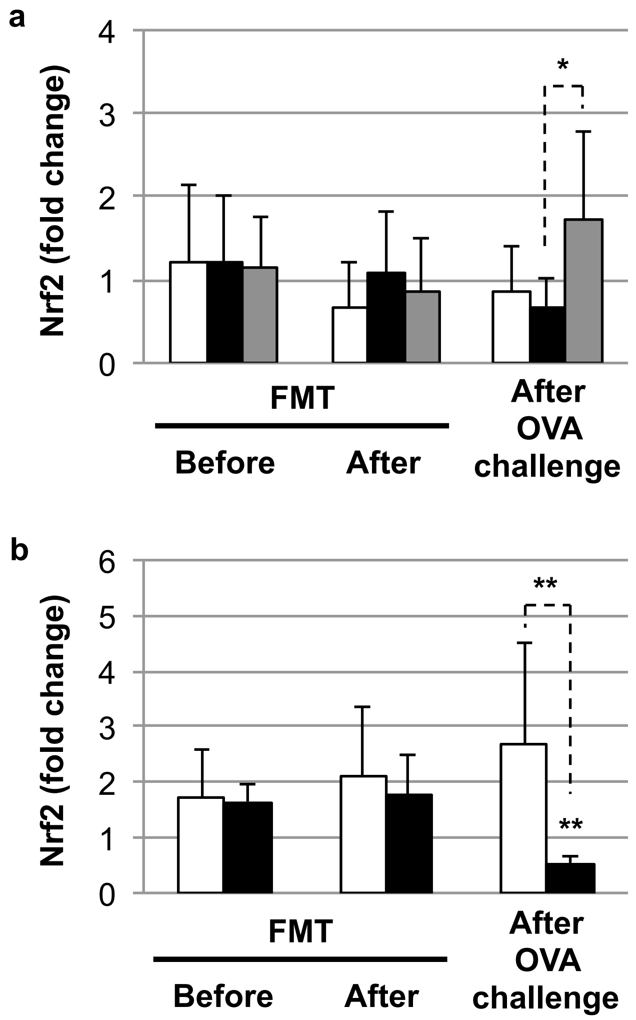

Supplementary Figure 7. Impact of fecal microbiota transplantation (FMT) on oxidative stress response in naïve and food allergy (FA) mice. (a) Naïve BALB/c mice received FMT with control donor feces (control-FMT, white bar, n=10), food allergy (FA) donor feces (FA-FMT, black bar, n=10) or phototherapy donor feces (phototherapy-FMT, gray bar, n=10). (b) FA mice received FMT with control donor feces (control-FMT, white bar, n=10). FA mice without FMT (black bar, n=5) were set as a comparison. Intestinal level of nuclear factor erythroid 2-related factor 2 (Nrf2) was evaluated by quantitative real-time PCR. *, **, $P < 0.05$ and 0.01 *vs.* baseline or the indicated pair, respectively (Student's *t*-test).

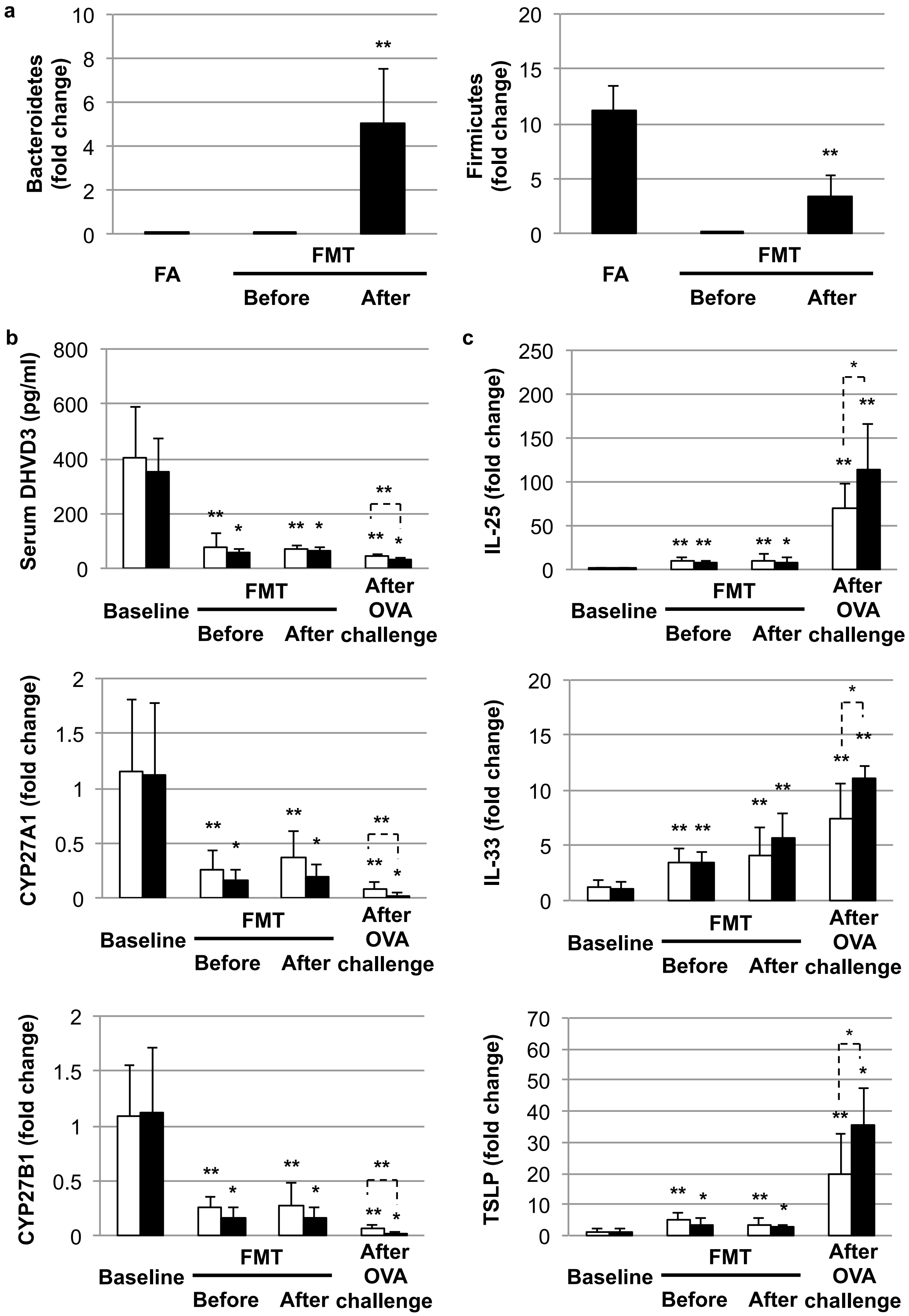
a
Bacteroidetes (fold change)
10
8
6
4
2
0
**
FA
FMT
Before
After
Firmicutes (fold change)
20
15
10
5
0
**
FA
FMT
Before
After
b
Serum DHVD3 (pg/ml)
800
600
400
200
0
Baseline
FMT
Before
After
After OVA challenge
CYP27A1 (fold change)
2
1.5
1
0.5
0
Baseline
FMT
Before
After
After OVA challenge
CYP27B1 (fold change)
2
1.5
1
0.5
0
Baseline
FMT
Before
After
After OVA challenge
c
IL-25 (fold change)
250
200
150
100
50
0
Baseline
FMT
Before
After
After OVA challenge
IL-33 (fold change)
20
15
10
5
0
Baseline
FMT
Before
After
After OVA challenge
TSLP (fold change)
70
60
50
40
30
20
10
0
Baseline
FMT
Before
After
After OVA challenge

Supplementary Figure 8. Impact of fecal microbiota transplantation (FMT) on the fecal microbiota composition, vitamin D_3 metabolisms and intestinal cytokine expression in food allergy (FA) mice. (a) FA mice received FMT with control donor feces (control-FMT, n=10). The phyla *Bacteroidetes* and *Firmicutes* were evaluated by quantitative real-time PCR. **, $P< 0.01$ *vs.* FA mice without FMT (n=5, Student's *t*-test). (b) Serum levels of vitamin D_3 or intestinal levels of sterol 27-hydroxylase (CYP27A1) and 25-hydroxyvitamin D_3-1α hydroxylase (CYP27B1) were evaluated by ELISA and quantitative real-time PCR, respectively. *, **, $P< 0.05$ and 0.01 *vs.* baseline or the indicated pair, respectively (Student's *t*-test). (c) Intestinal levels of IL-25, IL-33, thymic stromal lymphopoietin (TSLP) and nuclear factor erythroid 2-related factor 2 (Nrf2) were evaluated by quantitative real-time PCR. White bar: FA mice with Control-FMT, black bar: FA mice without FMT. *, **, $P<$ 0.05 and 0.01 *vs.* baseline or the indicated pair, respectively (Student's *t*-test).

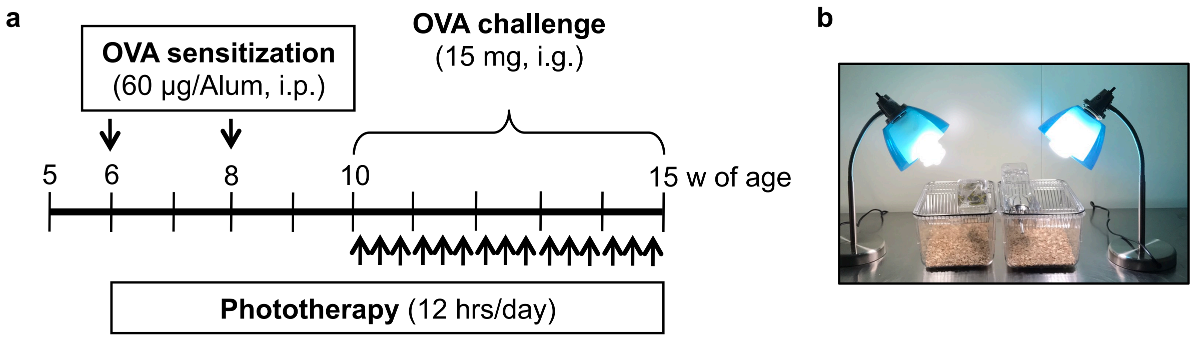

Supplementary Figure 9. Establishment of a food allergy (FA) model and phototherapy.

(a) Schedule for establishment of the FA model. BALB/c mice were intraperitoneally (i.p.) immunized twice with a precipitate (100 μl/mouse) of ovalbumin (OVA, 60 μg) and aluminum hydroxide (Alum, 1 mg) followed by repeated intragastric (i.g.) OVA (15 mg in a final volume of 150 μl of PBS) challenge 15 times. (b) Artificial sunlight setting for phototherapy. The phototherapy group received daily exposure to artificial sunlight (color temperature 5500 K, color rendition indexes >90) (12 hours/day) throughout the entire experiment (9 weeks).

Supplementary Table 1. Primer information.

Primer	Sequence
IL-25	Forward: 5'-ACAGGGACTTGAATCGGGTC-3'
	Reverse: 5'-TGGTAAAGTGGGACGGAGTTG-3'
IL-33	Forward: 5'-GGCTGCATGCCAACGACAAGG-3'
	Reverse: 5'-AAGGCCTGTTCCGGAGGCGA-3'
TSLP	Forward: 5'-CCAGGCTACCCTGAAACTGA-3'
	Reverse: 5'-TCTGGAGATTGCATGAAGGA-3'
VDR	Forward: 5'-TGACTTTGACCGGAATGTGCCT-3'
	Reverse: 5'-TTCATCATGCCAATGTCCACGCAG-3'
CYP27A1	Forward: 5'-CAACCTCCTTTGGGACTTAC-3'
	Reverse: 5'-TGATCCATGTGGTCTCTTATTG-3'
CYP27B1	Forward: 5'-CGCTAGTCTCCCTATGTCACTATGC-3'
	Reverse: 5'-AGCCGAAGGGAAGAGATGC-3'
Nrf2	Forward: 5'-CTGAACTCCTGGACGGGACTA-3'
	Reverse: 5'-CGGTGGGTCTCCGTAAATG-3'
HO-1	Forward: 5'-AGCACTATGTAAAGCGTCTC-3'
	Reverse: 5'-CGGTCTTAGCCTCTTCTGT-3'
SOD1	Forward: 5'-AACCAGTTGTGTTGTCAGGAC-3'
	Reverse: 5'-CCACCATGTTTCTTAGAGTGAGG-3'
SOD2	Forward: 5'-TGGACAAACCTGAGCCCTAAG-3'
	Reverse: 5'-CCCAAAGTCACGCTTGATAGC-3'
β-actin	Forward: 5'-GGCTGTATTCCCCTCCATCG-3'
	Reverse: 5'-CCAGTTGGTAACAATGCCATGT-3'

Supplementary Table 2. Primer information.

Primer	Sequence
Bacteroidetes	Forward: 5’-GAAGGTCCCCCACATTG-3’
	Reverse: 5’-CGCKACTTGGCTGGTTCAG-3’
Firmicutes	Forward: 5’-GGAGYATGTGGTTTAATTCGAAGCA-3’
	Reverse: 5’-AGCTGACGACAACCATGCAC-3’
Total bacteria	Forward: 5’-ACTCCTACGGGAGGCAG-3’
	Reverse: 5’-GTATTACCGCGGCTGCTG-3’
