# Supplementary material for: Daily full spectrum light exposure prevents food allergy-like allergic diarrhea by modulating vitamin D3 and microbiota composition
Source: NPJ Biofilms Microbiomes. 2021 May 6;7:41. doi: 10.1038/s41522-021-00213-8 (PMC8102508; doi:10.1038/s41522-021-00213-8)
Supplement: Supplementary file 2 — Reporting Summary [file 41522_2021_213_MOESM2_ESM.pdf]

nature research

Corresponding author(s): Toshiaki Nakano

Last updated by author(s): Feb 9, 2021

Reporting Summary

Nature Research wishes to improve the reproducibility of the work that we publish. This form provides structure for consistency and transparency in reporting. For further information on Nature Research policies, see our Editorial Policies and the Editorial Policy Checklist.

Statistics

For all statistical analyses, confirm that the following items are present in the figure legend, table legend, main text, or Methods section.

n/a	Confirmed	
☐	☒	The exact sample size (*n*) for each experimental group/condition, given as a discrete number and unit of measurement
☐	☒	A statement on whether measurements were taken from distinct samples or whether the same sample was measured repeatedly
☐	☒	The statistical test(s) used AND whether they are one- or two-sided<br>*Only common tests should be described solely by name; describe more complex techniques in the Methods section.*
☒	☐	A description of all covariates tested
☒	☐	A description of any assumptions or corrections, such as tests of normality and adjustment for multiple comparisons
☐	☒	A full description of the statistical parameters including central tendency (e.g. means) or other basic estimates (e.g. regression coefficient) AND variation (e.g. standard deviation) or associated estimates of uncertainty (e.g. confidence intervals)
☒	☐	For null hypothesis testing, the test statistic (e.g. *F*, *t*, *r*) with confidence intervals, effect sizes, degrees of freedom and *P* value noted<br>*Give P values as exact values whenever suitable.*
☒	☐	For Bayesian analysis, information on the choice of priors and Markov chain Monte Carlo settings
☐	☒	For hierarchical and complex designs, identification of the appropriate level for tests and full reporting of outcomes
☐	☒	Estimates of effect sizes (e.g. Cohen's *d*, Pearson's *r*), indicating how they were calculated

Our web collection on statistics for biologists contains articles on many of the points above.

Software and code

Policy information about availability of computer code

Data collection	No software was used.
Data analysis	FLASH (V1.2.7, http://ccb.jhu.edu/software/FLASH/)<br>QIIME (V1.7.0, http://qiime.org/index.html)<br>UCHIME algorithm (http://www.drive5.com/usearch/manual/uchime_algo.html)

For manuscripts utilizing custom algorithms or software that are central to the research but not yet described in published literature, software must be made available to editors and reviewers. We strongly encourage code deposition in a community repository (e.g. GitHub). See the Nature Research guidelines for submitting code & software for further information.

Data

Policy information about availability of data

All manuscripts must include a data availability statement. This statement should provide the following information, where applicable:

- Accession codes, unique identifiers, or web links for publicly available datasets
- A list of figures that have associated raw data
- A description of any restrictions on data availability

Raw data from the fecal microbiota analysis are deposited in the SRA database public repository from NCBI within the Bioproject accession number PRJNA690991.

April 2020

Field-specific reporting

Please select the one below that is the best fit for your research. If you are not sure, read the appropriate sections before making your selection.

☒ Life sciences ☐ Behavioural & social sciences ☐ Ecological, evolutionary & environmental sciences

For a reference copy of the document with all sections, see nature.com/documents/nr-reporting-summary-flat.pdf

Life sciences study design

All studies must disclose on these points even when the disclosure is negative.

Sample size	Power analysis (G*Power, http://www.gpower.hhu.de) was performed for sample size calculation.<br>We also check the reference (PLoS One 2012;7:e37156) to determine the sample size.
Data exclusions	No data were excluded from the data analyses.
Replication	We confirmed the reproducibility of the research findings.
Randomization	Animals were randomly divided into experimental groups.
Blinding	Group allocation/data collection and data analysis were performed by independent researchers.

Reporting for specific materials, systems and methods

We require information from authors about some types of materials, experimental systems and methods used in many studies. Here, indicate whether each material, system or method listed is relevant to your study. If you are not sure if a list item applies to your research, read the appropriate section before selecting a response.

Materials & experimental systems

n/a	Involved in the study	
☐	☒	Antibodies
☒	☐	Eukaryotic cell lines
☒	☐	Palaeontology and archaeology
☐	☒	Animals and other organisms
☒	☐	Human research participants
☒	☐	Clinical data
☒	☐	Dual use research of concern

Methods

n/a	Involved in the study	
☒	☐	ChIP-seq
☒	☐	Flow cytometry
☒	☐	MRI-based neuroimaging

Antibodies

Antibodies used	Mcpt-1 (Thermo Fisher Scientific, Cat No. 14-5503-82, clone RF6.1); IL-25 (Thermo Fisher Scientific, Cat No. MA1-41067, clone 68C1039.2); IL-33 (Thermo Fisher Scientific, Cat No. MA5-15773, clone 12H6D12); TSLP (Thermo Fisher Scientific, Cat No. PA5-20320); CYP27A1 (Thermo Fisher Scientific, Cat No. PA5-27946); CYP27B1 (Thermo Fisher Scientific, Cat No. PA5-79128); IgG1 (BD Biosciences, Cat No. 553440 and 553441, clone A85-1); IgG2a (BD Biosciences, Cat No. 553446 and 553388, clone R11-89 and R19-15); IgE (BD Biosciences, Cat No. 553413 and 553419, clone R35-72 and R35-118); Nrf2 (GeneTex, Cat No. GTX103322); β-Actin (Millipore, Cat No. MAB1501, clone C4)
Validation	Mcpt-1 (mouse, IHC); IL-25 (mouse, IHC); IL-33 (mouse, IHC); TSLP (mouse, IHC); CYP27A1 (mouse, IHC); CYP27B1 (mouse, IHC); IgG1 (mouse, ELISA); IgG2a (mouse, ELISA); IgE (mouse, ELISA); Nrf2 (mouse, WB); β-Actin (mouse, WB)

Animals and other organisms

Policy information about studies involving animals; ARRIVE guidelines recommended for reporting animal research

Laboratory animals	BALB/c mice, female, 4 weeks of age
Wild animals	The study did not involve wild animals.
Field-collected samples	The study did not involve samples collected from the field.
Ethics oversight	Kaohsiung Chang Gung Memorial Hospital

Note that full information on the approval of the study protocol must also be provided in the manuscript.
